# Decoding the intricate network of molecular interactions of a hyperstable engineered biocatalyst†

**DOI:** 10.1039/d0sc03367g

**Published:** 2020-09-11

**Authors:** Klara Markova, Klaudia Chmelova, Sérgio M. Marques, Philippe Carpentier, David Bednar, Jiri Damborsky, Martin Marek

**Affiliations:** Loschmidt Laboratories, Department of Experimental Biology and RECETOX, Faculty of Science, Masaryk University Kamenice 5 625 00 Brno Czech Republic jiri@chemi.muni.cz martin.marek@recetox.muni.cz; International Clinical Research Center, St. Anne's University Hospital Brno Pekarska 53 656 91 Brno Czech Republic; Université Grenoble Alpes, CNRS, CEA, Interdisciplinary Research Institute of Grenoble (IRIG), Laboratoire Chimie et Biologie des Métaux (LCBM) 17 Avenue des Martyrs 38054 Grenoble France; European Synchrotron Radiation Facility (ESRF) 71 Avenue des Martyrs 38043 Grenoble France

## Abstract

Computational design of protein catalysts with enhanced stabilities for use in research and enzyme technologies is a challenging task. Using force-field calculations and phylogenetic analysis, we previously designed the haloalkane dehalogenase DhaA115 which contains 11 mutations that confer upon it outstanding thermostability (*T*_m_ = 73.5 °C; Δ*T*_m_ > 23 °C). An understanding of the structural basis of this hyperstabilization is required in order to develop computer algorithms and predictive tools. Here, we report X-ray structures of DhaA115 at 1.55 Å and 1.6 Å resolutions and their molecular dynamics trajectories, which unravel the intricate network of interactions that reinforce the αβα-sandwich architecture. Unexpectedly, mutations toward bulky aromatic amino acids at the protein surface triggered long-distance (∼27 Å) backbone changes due to cooperative effects. These cooperative interactions produced an unprecedented double-lock system that: (i) induced backbone changes, (ii) closed the molecular gates to the active site, (iii) reduced the volumes of the main and slot access tunnels, and (iv) occluded the active site. Despite these spatial restrictions, experimental tracing of the access tunnels using krypton derivative crystals demonstrates that transport of ligands is still effective. Our findings highlight key thermostabilization effects and provide a structural basis for designing new thermostable protein catalysts.

## Abstract 2

Illustration of cooperative thermostabilization effects of the double-lock system that: (i) induced backbone changes, (ii) closed the molecular gates, (iii) reduced the volumes of the main and slot access tunnels, and (iv) occluded the active site.

## Graphical abstract

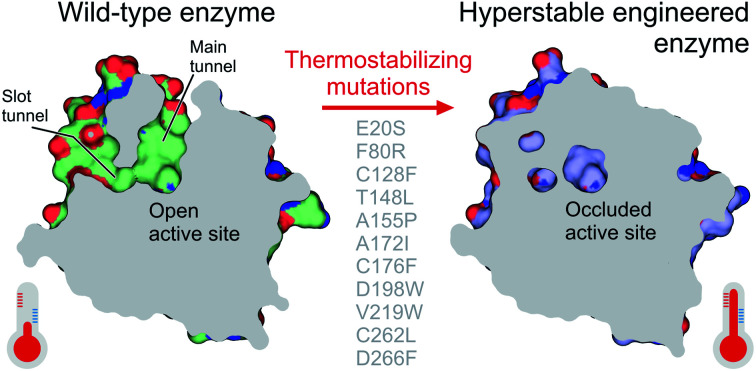

## Introduction

Enzymes have evolved for billions of years, and will continue to do so as long as life on earth exists.^1^ They catalyze almost all chemical reactions that occur in living organisms, and many of them have been successfully incorporated into diverse industrial, environmental and biomedical technologies.^2^ Often, wild type enzymes do not fully meet the demands of these harsh technological processes, and punctual mutations are engineered into them to improve their physico-chemical properties for technological applications. The key parameter for all enzymes to be employed in industrial catalysis is thermostability, which allows them to withstand elevated temperatures during biocatalytic processes.^3^

Enhancing protein thermostability involves changes that shift the folding–unfolding balance toward the folded form. Stabilizing substitutions can either stabilize the folded conformation or destabilize the unfolded one. The most direct way to stabilize proteins is to create or strengthen attractive interactions between amino acids in the folded conformation. Although proteins will continue to unfold anyway, these stronger interactions will either slow down unfolding or speed up refolding processes.^4^ A structured form can be stabilized through non-covalent interactions including hydrophobic interactions, hydrogen bonds, salt bridges and van der Waals forces.^5^ Increasing the number of stabilizing electrostatic interactions between residues of opposite charge reinforces proteins' thermal stability.^6^ Hydrophobic interactions have been shown to contribute proportionally more effectively to protein stability than hydrogen bonds.^7^ The hydrophobic effect is indeed the dominant driving force in protein folding, and designing a well-packed hydrophobic core is therefore usually an efficient strategy for engineering stable proteins.^4^

Haloalkane dehalogenases (HLDs; EC 3.8.1.5) are α/β-hydrolases that catalyze the hydrolytic cleavage of the carbon–halogen bond in diverse halogenated aliphatic hydrocarbons *via* S_N_2 nucleophilic substitution. The reaction requires the addition of a water molecule and releases a halide ion together with a proton, finally producing the corresponding alcohol.^8^ Structurally, HLDs consist of a canonical α/β-hydrolase fold, which is composed of a central eight-stranded β-sheet domain surrounded by several α-helices (*i.e.* αβα sandwich architecture). An additional versatile helical cap domain is observed to be specific to each HLD enzyme.^9^ The active site contains a catalytic pentad, which consists of a nucleophile, a base, a catalytic acid, and two halide-stabilizing residues.^9^ In all HLDs, the active site is positioned in a hydrophobic pocket buried between the α/β-fold core and the cap domain, and this catalytic center is connected with the bulk solvent *via* a main tunnel and a slot tunnel.^10^ Both of these tunnels are crucial determinants of the specific catalytic activity and the substrate selectivity of each HLD enzyme.^11,12^

Recently, we developed FireProt,^13,14^ a fully automated and robust computational pipeline combining energy- and evolution-based approaches to design highly stable multi-point mutant proteins. We employed FireProt to enhance the thermostability of DhaA, an HLD enzyme from *Rhodococcus rhodochrous* (*T*_m_ = 50.5 °C; *T*_opt_ = 45 °C). After several iteration cycles, we obtained an 11-point DhaA mutant, hereafter referred to as DhaA115, with outstanding thermostability (*T*_m_ = 73.5 °C) and thermophilicity, as demonstrated by a substantial shift in the optimal catalytic temperature (*T*_opt_ = 65 °C).^13^ Computational modeling showed that 3 of the 11 stabilizing residues line the main access tunnel, 3 other residues are buried within the protein core and the last 5 residues are exposed to solvent on the protein surface.^13^ We inferred that 8 of these mutations (C128F, T148L, A172I, C176F, D198W, V219W, C262L and D266F), which were identified by the energy-based approach, potentially enhance the stability of the enzyme by improving the packing of atoms within the protein interior and/or by strengthening hydrophobic interactions.^13^ However, the stabilizing effects of the 3 remaining mutations (E20S, F80R and A155P), proposed by the evolution-based approach, cannot be reproduced by force-field calculations.^15^ Experimental data are lacking to explain the structural basis for the engineered hyperstability of DhaA115.

To fill this gap, we crystallized and solved high-resolution structures of the hyperstable enzyme DhaA115. Analyses of these crystal structures highlight specific amino acid constellations that primarily reinforce the αβα-sandwich architecture and the helical cap domain *via* multiple newly-established interactions of the non-polar, hydrophobic and aromatic π–π stacking types. Surprisingly, we found that placement of bulky aromatic amino acids on the protein surface triggered some unexpected long-distance changes in the protein backbone. Essentially, these changes cause the gates and the internal volumes of both the main and the slot access tunnels to be restricted, and consequently the enzyme active site appears somewhat occluded. Interestingly, despite the active site occlusion, experimental mapping of the enzyme tunnels by krypton derivatization of the DhaA115 crystals, supported by protein dynamics simulations, showed that ligand molecules can still be transported through the enzyme tunnels. Collectively, our findings demonstrate that the hyperstabilization engineered in DhaA led to massive reduction in the volume of its access tunnels, and that the enzymes are still capable of operating since they are permeable to substrates, products and water molecules. This permeability is then increased at elevated temperature, as previously demonstrated by the shifted optimal catalytic temperature (*T*_opt_ = 65 °C) of the DhaA115 enzyme.^13^

## Results

### Crystal structure of the hyperstable enzyme DhaA115

To obtain precise structural information about how the DhaA enzyme is thermostabilized, we focused our efforts on crystallization of the most stabilized enzyme variant, DhaA115. We obtained crystals that belonged to the space group *P*12_1_1 and diffracted at 1.6 Å resolution (Table 1). The final model contains two enzyme molecules per asymmetric unit and has good values for deviation from the ideal (root mean-square deviation on the Cα atoms of 0.3 Å; Fig. S1†), with *R*-factor and *R*-free values of 0.16 and 0.18 respectively (Table 1). Almost all of the residues were built in density, except for a few residues at the disordered amino- and carboxy-terminal ends.

**Table 1:** Crystallographic data collection and refinement statistics

Data collection^a^	Native DhaA115	Krypton-soaked DhaA115
Wavelength (Å)	0.861	0.861
Space group	*P*12_1_1	*P*2_1_2_1_2_1_

Cell dimensions		
*a*, *b*, *c* (Å)	70.19, 68.12, 83.92	67.98, 82.04, 144.18
*α*, *β*, *γ* (°)	90, 104.82, 90	90, 90, 90
Resolution (Å)	48.07–1.6 (1.657–1.6)	49.2–1.55 (1.605–1.55)
Total reflections	673 251 (64 239)	1 562 749 (155 793)
Unique reflections	99 075 (9734)	116 063 (11 342)
*R*_merge_	6.85 (61.9)	6.3 (83.5)
*I*/*σI*	15.55 (2.55)	24.89 (3.45)
Completeness (%)	98.16 (97.14)	98.76 (98.12)
Multiplicity	6.8 (6.6)	13.5 (13.7)
CC(1/2)	99.9 (85.6)	100 (94.7)
Wilson *B*-factor	17.23	18.26

Refinement		
Resolution (Å)	48.07–1.6 (1.657–1.6)	49.2–1.55 (1.605–1.55)
No. reflections	99 075 (9733)	116 063 (11 335)
*R*_work_/*R*_free_	0.158/0.178	0.160/0.181

Number of atoms		
Protein	4837	4827
Ligand	62	125
Water	567	622

*B*-factors		
Protein	20.72	19.20
Ligand	31.74	31.56
Water	32.49	33.33

RMS deviations		
Bond lengths (Å)	0.006	0.006
Bond angles (°)	0.84	0.90
Ramachandran favored (%)	96.02	96.37
Ramachandran allowed (%)	3.98	3.63
Ramachandran outliers (%)	0	0
PDB ID code	6SP5	6SP8

a Values in parentheses are for the highest-resolution shell.

**Fig. 1:**
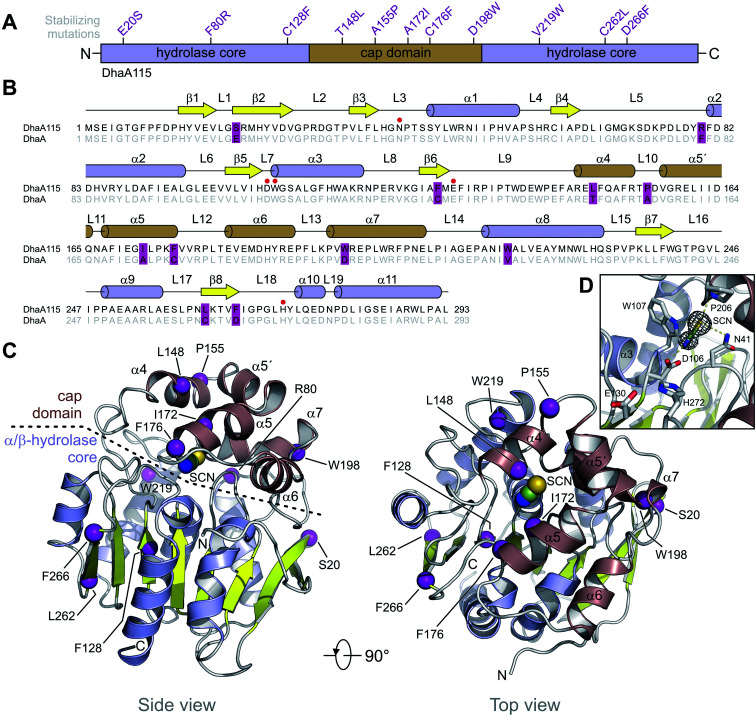
Overall structure of DhaA115. (A) Schematic representation of the protein sequence showing the domain topology of DhaA115 and the positions of the stabilizing mutations. (B) Structure-based sequence alignment of DhaA115 and DhaA. The stabilizing mutations are shown in violet frames. Secondary structure elements found in DhaA115 are shown above the alignment. Catalytically essential residues are pointed out with red dots. (C) Cartoon representation of DhaA115 structure with the central eight-stranded β-sheet (yellow), the α/β-hydrolase helices (blue) and the helical cap domain (brown). The stabilizing mutations are shown as purple spheres. The isothiocyanate (SCN) molecule bound in the enzyme active site is shown as spheres. (D) Close-up view of the enzyme's active site with key catalytically essential residues (grey sticks) and simulated annealing omit electron density map contoured at 2*σ* around the isothiocyanate (SCN) molecule. Molecular contacts between protein residues and SCN are shown as yellow dashed lines.

DhaA115 adopts a canonical HLD fold similar to that of the wild-type DhaA (RMSD on the Cα atoms of 0.6 Å; Fig. S2†), forming a single αβα sandwich architecture (α/β-hydrolase core) with a characteristic helical cap domain (Fig. 1). The α/β core is composed of a central eight-stranded β-sheet, with a β2 strand in an anti-parallel orientation. This α/β core is sandwiched by helices (α1–α3) on one side and (α8–α11) on the other. The helical (α4–α7) cap domain, which is positioned between the β6 strand and the α8 helix, shields the α/β-hydrolase core to which it is anchored *via* L9 and L14 loops. The enzyme active site is located in a predominantly hydrophobic cavity formed at the interface between the α/β-hydrolase core and the cap domain. The overall topology of the secondary structure elements is very similar to that of the wild-type DhaA. However, specific backbone re-arrangements are observed, which encompass the L9, L10 and L14 loops and the α4 and α9 helices (Fig. 1 and S2†).

Additionally, we unambiguously identified in the electron density map the presence of bis-tris propane (B3P), glycerol (GOL) and isothiocyanate (SCN) molecules, which were bound to the DhaA115 enzyme. Consistent with this, bis-tris propane and isothiocyanate were required in the crystallization solution, while the glycerol was used for cryo-protection. The bis-tris propane and glycerol molecules are bound on the protein surface, the former being also involved in crystal-packing contacts. There are three SCN-binding sites per enzyme molecule; two of them are also located on the enzyme surface while the last one is deeply buried in the active site cavity (Fig. S1†). As shown in Fig. 1D, the latter SCN anion interacts with three catalytic residues: the nucleophilic aspartate D106 (2.6 Å) and the two halide-stabilizing residues, W107 (3.5 Å) and N41 (3.6 Å). It is also in close contact with the non-catalytic proline P206 (3.3 Å). This SCN-binding site thus overlaps with the halide-binding site, where the halide anion product is usually captured during the dehalogenation reaction.

### Solution structure of the hyperstable DhaA115

**Fig. 2:**
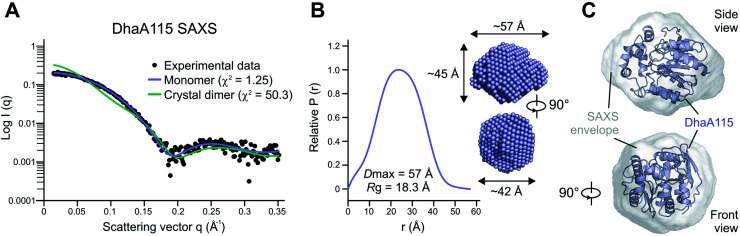
Solution structure of DhaA115 determined by SAXS. (A) Experimental SAXS scattering curve for DhaA115 (black dots) is shown against the calculated scattering curves for the DhaA115 monomer (blue line) and DhaA115 crystal dimer (green line). (B) Distance distribution function of DhaA115 computed from the X-ray scattering pattern using the GNOM program. (C) *Ab initio* molecular envelope generated from SAXS data analysis. The molecular SAXS envelope of the DhaA115 monomer is shown in a semi-transparent grey color superposed on the DhaA115 monomer of the crystal structure represented as a blue cartoon.

Whilst the wild-type DhaA is a monomeric enzyme, we previously noted that the stabilized DhaA115 variant forms a minority of dimers and high-molecular-weight oligomeric states.^15^ We therefore speculated as to whether the dimer observed in the asymmetric unit of the crystal (Fig. S1†) might also exist in solution. To test this hypothesis, we employed small-angle X-ray light scattering (SAXS) analysis to probe the DhaA115 structure in solution. The SAXS profile of the DhaA115 solution closely fits the scattering profile calculated using a single DhaA115 monomer of the crystal structure (*χ*^2^ = 1.25), but consistently does not correspond at all to the scattering curve calculated using the dimer of the crystal asymmetric unit (*χ*^2^ = 50.3; Fig. 2). Furthermore, the radius of gyration (*R*_g_) determined for the merged data has a value of 18.34 Å. The representative pair distance distribution function, *P*(r), evaluated by the indirect Fourier transform with the GNOM package,^16^ is shown in Fig. 2. The profile has a bell-like shape with a main peak at 23.4 Å, and trails off to a maximum dimension (*D*_max_) of ∼57 Å. Finally, the *ab initio* model reconstructed from the experimental SAXS data perfectly accommodates a monomer of the DhaA115 crystal structure (Fig. 2).

Our SAXS results demonstrate that the purified DhaA115 is indeed a monomeric enzyme. Complementary PISA calculations^17^ showed that the buried solvent-accessible area in the crystal contact DhaA115 dimer is ∼241 Å^2^, which represents only 2.1% of the total solvent-accessible surface area of the monomer (∼11 298 Å^2^). Taken together, the SAXS experiments and the PISA calculations provide evidence that the crystal contact dimer observed in the asymmetric unit (Fig. S1†) is not biologically relevant and does not exist in solution. Our data suggest that the DhaA115 dimers observed by Beerens and co-workers^15^ must employ a dimerization interface different from that observed in our crystal packing (Fig. S1†).

### Localization of the stabilizing mutations

Computer-aided design predicted eleven amino acid substitutions, whose simultaneous introduction into the DhaA enzyme resulted in a highly thermostable enzyme variant, DhaA115, with *T*_m_ = 73.5 °C and shifted optimal catalytic temperature (*T*_opt_ = 65 °C).^13^ As shown in Fig. 1A, the designed mutations are evenly distributed along the protein sequence, with 6 of them localized in the α/β-hydrolase core (E20S + F80R + C128F + V219W + C262L + D266F) and the remaining 5 mutations in the cap domain (T148L + A155P + A172I + C176F + D198W). Careful inspection of the DhaA115 structure revealed that nine out of the eleven mutations are located in the secondary structure elements (Fig. 1B), the other two (F80R + A155P) in the secondary structure/loop transitions.

### Structural implications of evolution-based mutations

**Fig. 3:**
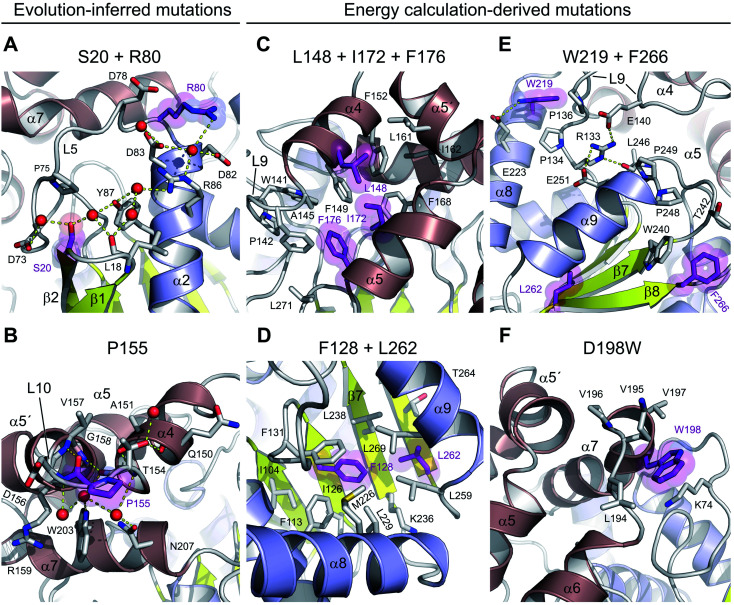
Stabilizing amino acid interactions observed in DhaA115. Close-up views of stabilizing residues and their interacting neighbours: S20 and R80 (A), P155 (B), L148, I172 and F176 (C), F128 and L262 (D), W219 and F266 (E) and W198 (F). The stabilizing residues are shown as purple sticks and semi-transparent spheres, the surrounding protein residues are shown as grey sticks, and water molecules as red spheres. Hydrogen bonds are shown as yellow dashed lines. The protein phylogeny-inferred mutations are in panels A and B, while the energy calculation-predicted mutations are in panels C–F.

There are three mutations that were designed by a protein evolution-based approach,^13^ namely E20S, F80R and A155P. All these residues are located on the enzyme surface (Fig. 1C), where they were found to either participate in the surface charge network important for protein–solvent interactions (Fig. 3A) or rigidify the solvent-exposed flexible loop (Fig. 3B). Specifically, the replacement of a surface phenylalanine with an arginine (F80R) disrupted the cation–π interaction between F80 and R204 (4.0 Å) present in the wild-type DhaA and established new ionic interactions with D78, D82 and D83. Moreover, there is a newly established water-mediated hydrogen-bonded network involving R80, D82, D83 and R86 (Fig. 3A).

Similarly, the serine residue (E20S) participates in the formation of an extensive solvent-mediated interaction network, in which the residues L18, S20, D73 and Y87 are involved. Strikingly, the water-mediated interactions between the L18, S20 and Y87 residues apparently rigidify the L1 loop connecting the β1 and β2 strands, and help to protect the central β-sheet (Fig. 3A). Both stabilizing S20 and R80 residues, which are located ∼16.5 Å apart from one another, participate extensively in local protein–water interactions, which contribute to the global solvent hydrogen-bonded network (Fig. 3A).

The last of the mutations deduced by the evolution-based approach is the substitution of an alanine by proline (A155P) in the L10 loop that connects the α4 and α5′ helices within the cap domain. This substitution forces the L10 loop to adopt a conformation different from that observed in DhaA. Specifically, the introduced proline residue (P155) is present in *trans*-conformation, which brings its carbonyl oxygen into a position where it can interact with the main-chain nitrogen of V157 (2.6 Å) (Fig. 3B). In addition, the new conformation of the L10 loop enables the molecule to establish two new main-chain to main-chain hydrogen bonds, namely between the carbonyl oxygen of T154 and the nitrogen of G158 (2.9 Å), and between the carbonyl atom of A151 and the nitrogen of T154 (3.1 Å). The L10 loop interacts extensively with an underneath α7 helix through multiple water-mediated hydrogen bonds in DhaA115, but not in DhaA (Fig. 3B), which again may have a positive effect on protein–solvent interactions.

### Structural implications of energy-based mutations

The remaining 8 mutations in DhaA115 (C128F, T148L, A172I, C176F, D198W, V219W, C262L and D266F) were inferred by force-field calculations.^13^ Interestingly, all these amino acids were mutations toward residues of the hydrophobic or aromatic type and always with a sterically bulkier side chain. Prior to our current work, these mutations were assumed to reinforce hydrophobic interactions and improve the protein packing.^13^ Our DhaA115 structure strongly confirms these assumptions, but we also see new structural effects that were not previously predicted by the computational design.

**Fig. 4:**
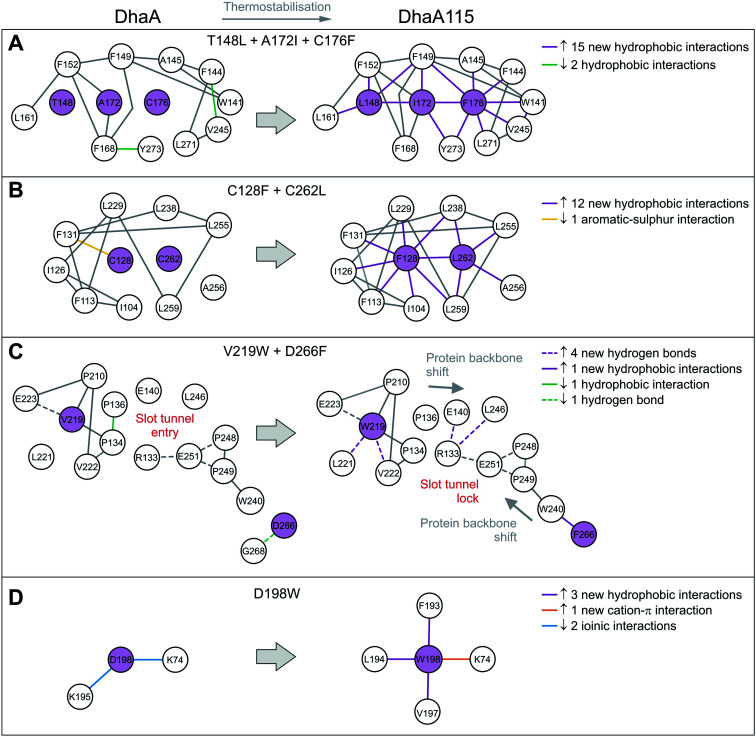
Selected residue–residue interaction networks in DhaA and DhaA115. (A–D) Panels depicting amino acid interactions networks for stabilizing mutations T148L/A172I/C76F (A), C128F/C262L (B), V219W/D266F (C) and D198W (D). The Protein Interactions Calculator (PIC) server was used to map residue–residue interactions. Amino acid residues that were mutated are shown in violet circles and the others in white. Newly established residue–residue interactions are depicted by violet and orange lines.

Crucially, we observe that the majority of the energy-based mutations (7 out of the 8, D198W being the exception) cooperate with each other and jointly contribute to the stabilization of the protein fold. Firstly, a triplet of mutations (T148L, A172I and C176F) localized in the cap domain interact with each other, but also strongly reinforce the hydrophobic and aromatic π–π interactions with the neighboring residues. These three stabilizing mutations interlock the α4, α5′ and α5 helices and adjacent L14 loop, thus rigidifying the top of the cap domain (Fig. 3C). Specifically, F176 forms a parallel-displaced stacking interaction with F149 (5.9 Å) and a T-shaped edge-to-face stacking interaction with F144 (5.8 Å), while L148 and I172 are involved in multiple hydrophobic and non-polar contacts with surrounding residues. Complementary calculations carried out by the Protein Interaction Calculator (PIC)^18^ identified 15 new hydrophobic interactions in DhaA115 due to this triple substitution. These interactions are not present in wild-type DhaA enzyme (Fig. 4A).

Similar cooperation takes place in the core of the enzyme with the substitutions C128F and C262L. The replacement of these two polar cysteine residues by the bulkier aromatic phenylalanine (F128) and the hydrophobic leucine (L262) enabled the formation of multiple new van der Waals contacts and non-polar interactions within the buried hydrophobic core of the α/β-hydrolase domain. The side chain of F128 interacts *via* a T-shaped edge-to-face stacking with F113 (5.2 Å), and *via* a Y-shaped stacking interaction with F113 (5.3 Å), forming the aromatic cage. Next to this, the mutated L262 residue complements a leucine-rich region that already includes L229, L238, L255 and L259 in wild type DhaA (Fig. 3D). Overall, our structure shows that F128 and L262 cooperatively contribute to tightening of the packing between the central β-sheet and the adjacent α-helical (α8 and α9) shell. The PIC calculations detected 12 newly-established hydrophobic contacts as a result of these interactions mediated by F128 and L262 (Fig. 4B).

Besides the short-distance cooperativities among the introduced mutations described above, we also observed long-distance cooperativity effects between mutations V219W and D266F. Interestingly, both substitutions are located on the protein surface, where the placing of the bulkier aromatic side chains of W219 and F266 triggered unexpected changes in the protein backbone (Fig. 3E). Specifically, these hydrophobic residues tend to minimize solvent exposure, and to compact the protein fold *via* interactions with amino acids buried in their surroundings. First, the side chain of W219 adopts a flipped-in conformation, which enables its indole nitrogen to be hydrogen-bonded with the carboxyl group of E223. More importantly, the flipped-in conformation of W219 has triggered a major structural re-arrangement of the L9 loop (Fig. 3E). These changes are accompanied by the formation of a new network of interaction between residues R133, E140, E251 and L246. This new re-arrangement is further favored by a slight tilting (∼7°) of α9 helix indirectly induced by F266 and to a lesser extent by L262 (Fig. 3E). The bulkier mutated F266 (β9) pushes the side chain of W240 (β7) toward the α9 helix which tilts, then this slight reorientation allows R133, E140, E251 and L246 to interact with each other (Fig. 3E). Our analysis has unraveled an epistatic interaction network by which the simultaneous substitutions of two remote residues (∼27 Å) on the protein surface (V219W and D266F) can trigger long-distance structural re-arrangements (Fig. 3E and 4C).

Finally, the last energy calculation-derived mutation, D198W, substitutes an aspartate that forms two ionic interactions with K74 and K195 in DhaA. However, its replacement with the bulky aromatic tryptophan (D198W) established a strong cation–π interaction with K74 (4.7 Å) and three additional new hydrophobic contacts with F193 (4.1 Å), L194 (4.8 Å) and V197 (3.8 Å) (Fig. 3F and 4D).

### Thermostabilization induced unexpected changes in the protein backbone

**Fig. 5:**
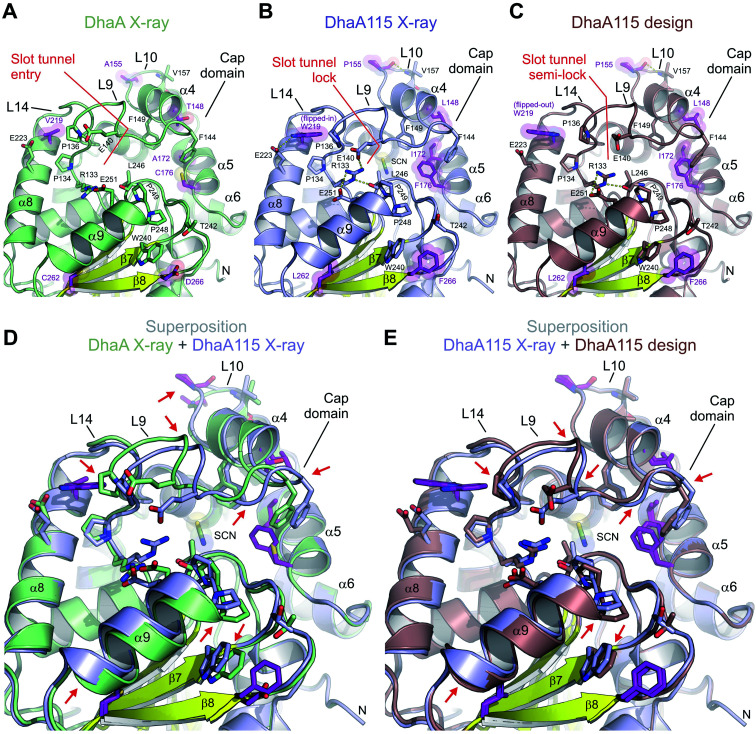
Cooperative effects of distant surface bulky side chains in slot tunnel closure. (A–C) Close-up cartoon representations of X-ray structure of DhaA (A), DhaA115 computational design (B) and DhaA115 X-ray structure (C). Note that in DhaA (A) the slot tunnel entry is not blocked by any residues, while in both the DhaA115 X-ray structure (B) and the DhaA115 design (C) it is markedly impaired. This is most pronounced in the X-ray structure, where the residue W219 adopts a flipped-in conformation leading to a major structural re-arrangement of the L9 loop, which, together with the α9 helix and L16 loop, forms the so-called slot tunnel lock (C). The stabilizing residues are shown as purple sticks and semi-transparent spheres. Hydrogen bonds are shown as yellow dashed lines. (D and E) Structural superposition of DhaA X-ray on DhaA115 X-ray (D) and DhaA115 X-ray on DhaA115 design (E). The stabilizing residues are shown as purple sticks. Red arrows depict major structural changes in the backbone. Note that the stabilizing mutations V219W and D266F induced structural re-arrangements of both L9 and the α9 helix, leading to the closing of the slot tunnel.

We show that the computer-aided thermostabilization of DhaA *via* 11 point mutations (DhaA115) not only affected the corresponding side-chain to side-chain and/or side-chain to main-chain interactions, but also induced major protein backbone changes (Fig. 5 and S2†). There is almost perfect agreement between the computational design and the crystallographic structure of DhaA115 in the re-arrangement of the L10 loop. The DhaA115 crystallographic structure revealed that the introduced proline (P155) adopts a *trans*-conformation leading to structural re-arrangement of the L10 loop, and confirmed the prediction of the protein design (Fig. 3B and 5E).^13^

The major discrepancies between the designed and X-ray structures of DhaA115 consist in different structural organizations encompassing the L9 and L14 loops, and the α4 and α9 helices. Careful inspection of all structural models – X-ray DhaA template, Dha115 design and X-ray Dha115 – provided an explanation for these dissimilarities (Fig. 5). Firstly, the substitution of a relatively small valine with a bulky and aromatic tryptophan (V219W) triggers the major structural change in the L9 loop. This L9 re-arrangement is most pronounced in the DhaA115 X-ray structure, where the W219 is observed to adopt a flipped-in conformation, which then forces the L9 loop to re-arrange substantially (Fig. 5B). However, a different conformation was observed in the predicted DhaA115 design structure, where the corresponding W219 adopts a flipped-out orientation, which is not likely to exert analogous steric pressure on the L9 loop to re-arrange to the same extent as that observed in the DhaA115 X-ray structure (Fig. 5C). Moreover, the structural re-arrangement of the L9 loop occurred concomitantly with a slight tilt of the α4 helix (∼6.3°), which tightly presses against the opposing α5 helix in the DhaA115 X-ray structure (Fig. 5D and S3†). Secondly, our DhaA115 X-ray structure shows that the aspartate-to-phenylalanine substitution (D266F) triggers structural changes that are more severe than those predicted. As shown in Fig. 5, the presence of the bulky side chain of F266 indirectly, through interaction with W240, displaced the α9 helix toward the slot tunnel entry. Here, it is important to note that another introduced mutation, L262, is also likely responsible for the displacement of the α9 helix (Fig. 5). As a result, several residues lining the slot tunnel entry, especially R133, E140, E251 and L246, are dramatically re-arranged, and form multiple new hydrogen bonds, creating a so-called slot tunnel lock in the crystal structure of DhaA115 (Fig. 5).

Our observations point to the fact that the stabilizing mutations may substantially affect the enzyme access tunnels that connect the active site with the bulk solvent. These tunnels are functional and ensure proper transport of substrate and product molecules to and from the active site, and are known determinants of catalytic properties for this enzyme family.^9^

### Thermostabilization reduced the volume of the enzyme access tunnels

**Fig. 6:**
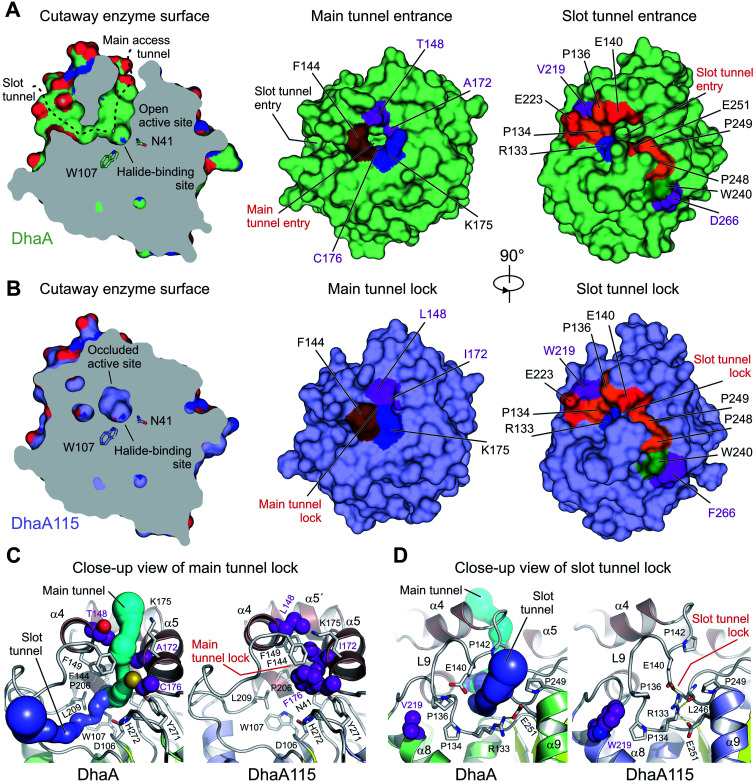
Visualization of access tunnels. Cutaway surface representations of enzyme active site and access tunnels for the wild type DhaA (A) and the hyperstable DhaA115 (B) enzymes. Note that the DhaA enzyme has a so-called open active site connected with the outside bulk solvent *via* both the main and slot access tunnels. In contrast, in DhaA115 the main and slot access tunnels are to a large extent blocked because of the direct or indirect effects of stabilizing mutations. Visualization of the CAVER-calculated main (C) and slot (D) access tunnels. The main tunnel was calculated (cyan) for wild type DhaA (left panel). The tunnel is absent in DhaA115 because of three stabilizing residues (L148, I172 and F176) which, together with other hydrophobic and aromatic residues (F144, F149 and L209) form the so-called main tunnel lock (right panel). Similarly, the slot tunnel calculated (blue) for wild type DhaA (left panel) is absent in DhaA115 because of a conformational change of the L9 loop triggered by the presence of stabilizing residue W219 and a positional shift of the α9 helix. This new constellation allows the residues R133 and E140 to form a so-called slot tunnel lock (right panel). The stabilizing residues are shown as purple spheres, and the other protein residues are shown as grey sticks.

The buried active site of the wild-type DhaA enzyme is connected with the bulk solvent *via* two tunnels, namely the main p1 access tunnel and the slot p2 access tunnel (Fig. 6). It has been previously shown that engineering of one of these tunnels may yield enzyme variants with modified substrate preference, enantioselectivity and thermostability.^12,19,20^

Analysis of the enzyme access tunnels and the active site cavity in DhaA115 revealed that the volumes of both the main and the slot access tunnels are greatly reduced, and that the enzyme active site is occluded (Fig. 6). Unlike in DhaA, CAVER calculations on the static X-ray DhaA115 structure did not detect any tunnels with minimum radius above 0.9 Å (Fig. 6C and D). Careful inspection of the DhaA115 crystal structure revealed that the main access tunnel is blocked by the triplet of stabilizing residues, namely L148, I172 and F176. The tight packing of these residues with a few neighboring residues (F144, F149 and K175) is the major hallmark of a main tunnel lock (Fig. 6C).

While partial closure of the main access tunnel was previously observed in several engineered DhaA variants,^12,19^ the simultaneous closure of both the main and the slot tunnels is a unique feature seen for the first time in DhaA115. This double-lock is enabled by: (i) the triplet of residues (L148, I172 and F176), coupled with the re-positioning of the α4 helix, that lock the main access tunnel and (ii) the structural re-arrangement of the L9 loop coupled with the re-positioning of the α9 helix, which lock the slot access tunnel. As described above, we show that residues W219, L262 and F266 are key drivers of these latter structural re-arrangements, which bring the residues R133, E140, L246 and E251 close to each other to create the slot tunnel lock (Fig. 5B and 6D).

### Experimental tracking of the access tunnels using krypton

Despite the fact that the crystal structure of DhaA115 showed a greatly reduced volume for both enzyme access tunnels, it has been previously shown that this hyperstable enzyme (*T*_m_ = 73.5 °C) still possesses dehalogenase activity, with a shifted optimal catalytic temperature (*T*_opt_ = 65 °C). We therefore aimed at experimental mapping of the enzyme access tunnels to test whether small molecules can still be transported to the active site of DhaA115. We employed a “soak-and-freeze” method that allows crystals to be processed in a pressurized krypton atmosphere.^22^ The DhaA115 crystals were soaked in krypton at a pressure of 150 bar, thereafter flash-frozen while still under high pressure, and then recovered in liquid nitrogen, in which the derivatives are stable for X-ray data collections. Anomalous diffraction data were collected at the krypton X-ray absorption edge (a wavelength of 0.861 Å).

Interestingly, soaking DhaA115 crystals in a high-pressure krypton atmosphere yielded crystals with a higher symmetry space group, *P*2_1_2_1_2_1_, and they diffracted to 1.55 Å resolution (Table 1). The final structural model of the krypton derivative of a DhaA115 crystal again revealed two enzyme molecules in the asymmetric unit (RMSD on Cα′s of 0.2 Å; Fig. S4†), and had good values for deviations from the ideal geometry, with *R*-factor and *R*-free values of 0.16 and 0.18 respectively (Table 1). Importantly, krypton derivatization did not induce any structural changes in the protein backbone, and superposition with the native DhaA115 showed an RMSD for the Cα atoms of only 0.2 Å (Fig. S5†).

**Fig. 7:**
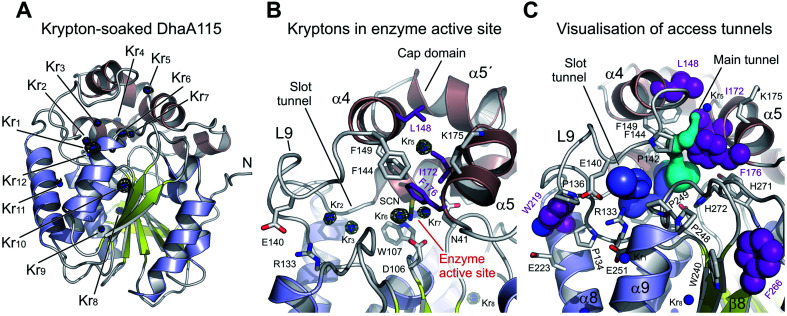
Experimental tracking of the enzyme access tunnels. (A) Cartoon representation of DhaA115 krypton derivative crystal structure with the central eight-stranded β-sheet (yellow), α/β-hydrolase helices (blue) and helical cap domain (brown). The locations of the krypton atom sites (Kr_1_ to Kr_11_) in pressurized DhaA115 are shown as blue spheres with anomalous Fourier maps contoured at 4*σ* (green mesh). (B) Close-up view of the active site and access tunnels in the DhaA115 krypton derivative crystal structure. Two krypton atoms (Kr_6_ and Kr_7_) are found in the enzyme active sites, two (Kr_2_ and Kr_3_) occupy the slot tunnels, and one krypton (Kr_5_) sits on top of the cap domain. The stabilizing residues are shown as purple sticks and the other protein residues are shown as grey sticks. (C) Visualization of the access tunnels in the DhaA115 krypton derivative crystal structure calculation by CAVER. The main (cyan) and slot (blue) tunnels are partially restored upon pressurization. The main tunnel is depicted in cyan and the slot tunnel in light blue. The stabilizing residues are shown as purple spheres, remaining residues are shown as grey sticks, and krypton atoms as blue spheres.

The derivatized DhaA115 structure shows 12 binding krypton sites (Kr_1_–Kr_12_) per enzyme molecule (Fig. 7 and S4†). Two krypton atoms (Kr_6_ and Kr_7_) are found in the predominantly hydrophobic cavity of the enzyme active site, and in close proximity to the isothiocyanate (SCN) bound in the halide-binding site. Two additional krypton atoms (Kr_2_ and Kr_3_) occupy the slot tunnel, and one krypton atom (Kr_5_) sits in the entrance of the main tunnel entry (Fig. 7B). Three krypton atoms (Kr_8_, Kr_9_ and Kr_11_) are bound in separate internal hydrophobic cavities, far away from the active site. The remaining four krypton atoms (Kr_1_, Kr_4_, Kr_10_ and Kr_12_) are found at the surface of the protein, occupying excavations of moderate hydrophobicity or mediating crystal packing contacts.

Finally, tunnel calculations on the krypton-soaked DhaA115 structure detected partial restoration of both main and slot tunnels (Fig. 7C), although these tunnels are still much reduced as compared to those observed in the wild-type DhaA enzyme (Fig. 6). Below we compare these results with a dynamical overview of the tunnels. Our structural data demonstrate that the engineered hyperstabilization led to massive reductions in the volumes of both enzyme access tunnels, but also that these latter are still permeable for small molecules during catalysis. These observations suggest that permeability is likely increased with elevated temperature, as previously shown by the shift in the optimal catalytic temperature (*T*_opt_ = 65 °C) of DhaA115.^13^

### Analysis of structure flexibility by molecular dynamic simulations

The structure of DhaA115 was subjected to molecular dynamics (MD) analysis to assess its intrinsic flexibility and to compare it with that of the wild type DhaA, a study which was reported previously.^15^ The physical conditions at which the MDs were performed, namely 310 K (37 °C), pH 7.5, and salt concentration of 0.1 M, correspond to the conditions at which HLDs are typically characterized in terms of activity and specificity.^11,12^ These simulations, run in duplicate for a total time of 200 ns, were properly equilibrated and converged (Fig. S6A†). The MDs showed that the backbone of DhaA115 was similarly rigid to, or slightly more rigid than, that of DhaA (Fig. S7A†). The main difference was around residues 31 and 155, which presented considerably higher *B*-factors in the wild-type DhaA than in the stabilized mutant.

Accelerated MDs (aMDs) were also carried out. The aMD technique is an enhanced-sampling method that applies a boost of potential energy that raises the energy of local minima and thus decreases the energy barriers, resulting in higher conformational transition rates. This method can be useful for better sampling the dynamic behavior and the conformational space of biomolecular systems over longer time-scales.^23,24^ These aMDs were well equilibrated and stable (Fig. S6B†). The *B*-factors were higher in the aMDs than in the classical MDs, and this was expected from simulations that promote higher conformational diversity (Fig. S7B†). Moreover, in the long run, only the region around residue 31 remained more rigid in DhaA115 than in the wild-type DhaA, and the rest of the protein showed similar flexibility. The region around residue 77 seems to be slightly, but not significantly, more flexible in the mutant than in the wild-type enzyme.

We also aimed to assess whether the structural changes induced by the mutations that were seen in the crystal structures are also maintained during MD simulations. As expected, both DhaA115 and DhaA relaxed from their respective crystallographic conformations after the equilibration steps. The differences were minimal, with RMSD slightly higher for the wild-type DhaA than for the DhaA115 mutant (Fig. S6 and Table S1†). One of the main differences previously highlighted was the flipped-in conformation of the mutated residue W219. During all simulations this residue maintained such an orientation and showed very low deviations from the DhaA115 crystal structure (RMSD in aMDs = 0.88 ± 0.28 Å; Table S1†), with similar values as observed for V219 in DhaA (RMSD in aMDs = 0.82 ± 0.27 Å). When we analyzed the potential effect of W219 on loop L9, we observed that the initial distances between W219 and P134 or W219 and P136 (both prolines being localized in the L9 loop) were slightly increased during the simulations. Our results thus suggest that the crystal packing had some influence on shortening these distances as compared to the average structures in solution (*d*_1_–*d*_3_ distances in Table S1†). However, the distances from W219 to the L9 loop were significantly longer in DhaA115 than in the case of V219 in DhaA, indicating that the effects of W219 in levering up loop L9 were preserved during MD simulations.

Another unexpected backbone change in α9 helix positioning observed in the DhaA115 crystal structures is due to the D266F mutation. The distances from D266/F266 (located in the β8 strand) to the backbone of W240 (in the β7 sheet) are very stable and close to those in the crystal structures, and the conformation of this region is not especially different from the wild-type DhaA (distance *d*_4_ in Table S1†). However, the average distances between the α9 helix and β7 sheet (*d*_5_) and between the α9 helix and β8 sheet (*d*_6_) remained greater in the simulations of DhaA115 as compared to DhaA (Table S1†). These MD observations support the crystallographic findings that the protein backbone was unpredictably re-arranged due to the thermostabilization process. We also verified the effects on the structural rearrangement at the mouth of the slot tunnel, presumably locked through an extended hydrogen bond network (Fig. 5B). The residues R133, E140, E251 and R254 were making H-bonds and were intermittently in contact with one another. In spite of the initial difference in the crystal structure of DhaA for E140, during the simulations the average distances between these four residues were very similar for DhaA115 and DhaA, and consistent with a true hydrogen-bond network (distances *d*_7_–*d*_9_ in Table S1†).

### Tunnel properties in molecular dynamic simulations

The access tunnels were calculated during the MD simulations using CAVER 3.02.^21^ This allowed us to assess how their geometry and potential relevance changed in comparison with the static crystal structures. We found that the tunnels in DhaA115 were still very narrow during the MD simulations (Table S2 and Fig. S8 and S9†), with no tunnels detected for the selected probe size (0.7 Å) in a large fraction of the trajectories. When tunnels were detected, they still had very low bottleneck radii (BR-value) most of the time. For example, the most defined tunnel, p2b, showed an average BR-value of 0.84 ± 0.11 Å. Very importantly, however, these tunnels were able to occasionally open up to significant values, *e.g.* tunnels p2b and p1 showed maximum BR-values of 1.34 and 1.41 Å.

The krypton-derivatized DhaA115 crystal structure was also simulated (RMSD plots in Fig. S6†), and its tunnels showed behavior very similar to those in the native DhaA115 structure, in terms of the preferred tunnels and their main properties. As expected, this analysis showed that krypton-derivatization did not alter the natural dynamical properties of DhaA115. When we looked at the aMDs, the access tunnels of DhaA115 fluctuated more in terms of radius, length and topology (larger standard deviations from the mean values). Such behavior could be expected from a simulation that promotes conformational transitions, such as aMD. The tunnels detected here also had larger average BR-values than in the MDs, but more importantly, they displayed considerably larger BR-value maxima across the simulations (tunnels p4 and p1 had maximal BR-values of 1.69 and 1.61 Å respectively). With bottleneck radii of this magnitude, these access tunnels can be considered to be open to the transit of solvent and small ligands (1.4 Å is the minimum radius required for a water molecule to pass through). These values are significantly larger than the size of the access tunnels found in the crystal structures and demonstrate the importance of statistical analysis of tunnels in dynamics rather than drawing conclusions based solely on a single static crystallographic structure. Our results show that although DhaA115 has a well-packed structure with an occluded active site pocket, it is still able to open occasionally and allow the transport of substrates and products. These findings explain the ability of DhaA115 to catalyze the dehalogenase reaction.

Finally, from a comparison of DhaA115 with the wild-type DhaA and DhaA31, another active DhaA variant bearing mutations that significantly narrowed its tunnels,^12^ DhaA115 has the narrowest tunnels, both in the respective crystal structures and in the MD simulations. Another major difference among the variants is in the topology of the relevant tunnels. While for DhaA and DhaA31, the most important was the main p1 tunnel, the slot p2 tunnel became more structurally relevant in DhaA115.

## Discussion

Improving enzyme stability is one of the major tasks in contemporary protein engineering. Many computational tools have been developed to make rational predictions of the effects of mutations on protein stability.^13,25–27^ In-depth structural understanding of these effects can help improve the accuracy of computer algorithms.

Several distinct strategies have been employed to stabilize proteins of the α/β-hydrolase fold family, namely: (i) structure-based computational approaches and informed mutagenesis of flexible regions, (ii) sequence-based phylogenetic approaches, and (iii) randomized mutagenesis coupled with extensive library screening.^28^ Generally, stabilizing mutations have been found to occur in both the cap and the catalytic domains, in buried regions and surface-exposed areas.^28^ For instance, *de novo* engineering of a disulfide bond physically anchoring the cap domain to the catalytic α/β-hydrolase domain was successfully used for stabilization of lipases,^29–31^ acetylcholine esterase^32^ and the haloalkane dehalogenase DhlA.^33^ Moreover, mutations leading to enhanced interior packing have been reported,^34–36^ as well as mutations introduced to decrease flexibility and increase stability of the α/β-fold proteins.^37–39^ Plant esterase was stabilized to resist heat inactivation by introducing proline residues into solvent-exposed loops.^40^ Several groups reported achieving increased stability of lipases through single point substitutions enabling the formation of new ionic interactions and salt bridges.^41–44^ It was previously shown that narrowing or blocking access tunnels helps to stabilize enzymes with buried active sites and to increase their resistance to organic co-solvents.^19,45^ Re-engineering of the access tunnels through five point mutations increased both catalytic activity toward 1,2,3-trichloropropane and thermal stability in DhaA31.^12^ Almost complete closure of the main tunnel while preserving the slot tunnel was observed in the stabilized mutant DhaA80 (*T*_m_ = 64.5 °C).^19^

The in-house FireProt server is an automated computational tool combining energy- and evolution-based approaches to design highly heat-stable mutants.^13^ The 11-point mutant haloalkane dehalogenase DhaA115, designed by FireProt, has the highest thermostability of all the DhaA variants ever engineered DhaA. However, the structural basis of this hyperstability was poorly understood. In this work we solved the high-resolution structures of DhaA115 and compared them with those of the wild type DhaA. Careful inspection of the DhaA115 structure revealed that 9 out of the 11 stabilizing mutations are located in the secondary structure elements. The mutations designed by an evolution-based approach (E20S, F80R and A155P) participate extensively in the surface charge network, protein–solvent interactions and/or rigidifying a solvent-exposed loop. We therefore conclude that newly-established protein–solvent interactions on the protein surface might be important factors in protecting the α/β-hydrolase core to stabilize the overall protein fold. Our structural data are thus in agreement with previous observations by Beerens and co-workers,^15^ who have shown experimentally that stabilization by evolution-based mutations is driven by both entropy and enthalpy, the former being difficult to predict from force-field calculations.^13^ Computational prediction tools such as FoldX^26^ and Rosetta^27^ do not evaluate entropic contributions correctly due to underestimating key factors such as alternative protein conformations and specific interactions between a protein and solvent molecules.^4^

The remaining 8 mutations (C128F, T148L, A172I, C176F, D198W, V219W, C262L and D266F) were inferred by an energy-based approach.^13^ It has been proposed that these mutations should reinforce hydrophobic and aromatic interactions and improve protein packing. In general, our experimentally determined DhaA115 structures confirm this proposal. We observe that the vast majority (7 out of 8) of the mutations cooperate with one another, showing effects on residue-to-residue packing and on stabilization of the protein fold. All energy-deduced mutations are hydrophobic or aromatic (always sterically bulkier than the original residues); however this led to unexpected structural effects. We reveal that the replacement of smaller residues with amino acids with bulkier side chains, *e.g.*, V219W, C262L and D266F, leads to long-distance re-arrangements of the protein backbone, which were not predicted in the original computational design. The backbone rearrangements remained stable during the MD simulations. These unexpected structural effects led to the production of the so-called double-lock system: (i) they closed the active site access gateways, (ii) the volumes of both main and slot enzyme tunnels were reduced, and (iii) the active site was occluded. We think that the restricted tunnels are likely the major determinant of the lower activity of DhaA115 at a temperature optimal for DhaA.^13^ Experimental tracking of the tunnels by krypton-derivatization of DhaA115 crystals, supported by protein simulations, revealed that ligands can still be transported through the tunnels as they can open to a significant extent. We expect that this tunnel opening will be even more pronounced at higher temperatures, which would explain the shift in the temperature optimum to a higher range.

Taken together, our experimental and theoretical results provide molecular insights into the engineered stability of DhaA115 and the impact of introduced mutations on functionally important structural features of this hyperstable enzyme. Our data pave the way for similar engineering efforts to be applied to various protein catalysts from the α/β-hydrolase family, but also to other structurally unrelated protein folds. Importantly, understanding of the structural basis of thermal stability in a protein designed by force-field calculations and phylogenetic analysis provides valuable information for further improvement of algorithms and computational workflows for achieving protein stabilization by rational protein design.^46^ One of the lessons learned from the structural analysis reported in this study is that the accumulation of experimentally verified single-point mutations will not lead to the structural basis of stabilization observed with DhaA115. Multiple substitutions must be introduced simultaneously to achieve cooperative effects, like backbone changes, sealing of auxiliary access tunnels, and formation of occluded active sites. Computational tools predicting the multiple substitutions, such as FireProt^13,14^ and PROSS^47^ are already available for this type of design. However, there is a space for further improvement of these hybrid protein stabilization platforms. Computational design of protein tunnels is underexplored strategy,^48,49^ which can be supported by the tools for calculation of access tunnels (*e.g.*, CAVER^21^) and ligands' passage (*e.g.*, CaverWeb^50^). The development of novel algorithms and software tools for rational engineering of protein loops is highly desirable, but still challenging. New experimental data^51^ and better understanding of structure–stability relationships are also essential premises for developing more reliable predictive models by machine learning.^52^

## Experimental methods

### Protein expression and purification

The DhaA115 enzyme was overproduced in *Escherichia coli*, as previously described. Briefly, DhaA115 was overexpressed in *E*. *coli* BL21(DE3) with induction by 0.5 mM IPTG at 20 °C for 16 hours. The cells were harvested by centrifugation at 11 806*g* at 4 °C for 10 min. The pellet was re-suspended in purification buffer A (500 mM NaCl, 10 mM imidazole, 20 mM potassium phosphate buffer pH 7.5) and sonicated using a Sonic Dismembrator Model 705 (Fisher Scientific, USA) in 3 cycles, each of 2 min (5 s pulse/5 s pause) with amplitude 50%. Disrupted cells were centrifuged at 21 000*g* at 4 °C for 1 h. His-tagged DhaA115 protein was purified on a Ni-chelating column (Ni-NTA Superflow cartridge) equilibrated in purification buffer A. The affinity-purified enzyme was eluted by a purification buffer A supplemented with 300 mM imidazole. The eluted protein was further purified by size-exclusion chromatography on a HiLoad 16/600 Superdex 200 gel filtration column (GE Healthcare) equilibrated in GF buffer (50 mM NaCl, 10 mM Tris pH 8.0). Peak fractions were pooled and concentrated with an Amicon Ultra centrifugal filter unit (Merck Millipore Ltd) to a final concentration of 11.5 mg ml^−1^. Protein concentration was measured on a DeNovixR^®^ DS-11 Spectrophotometer (DeNovix Inc., USA).

### Crystallization, krypton-soaking and diffraction analysis

Diffraction-quality crystals of the DhaA115 enzyme were obtained at 20 °C by mixing equal volumes of DhaA115 (11.5 mg ml^−1^) with reservoir solution composed of 18–24% PEG 3350, 0.2 M KSCN and 0.1 M bis-tris propane (pH 6.5), and crystallized using the hanging-drop vapor diffusion technique. After 3–6 days, the crystals so grown were briefly transferred into reservoir solution supplemented with 22% glycerol and flash-frozen in liquid nitrogen.

Krypton derivatives were produced using ‘soak and freeze’ methodology.^22^ The method is aimed at deciphering functional tunnels in proteins.^53,54^ In practice, a crystal obtained from 1 : 1 protein (13.8 mg ml^−1^)/reservoir solution was fished out into a capillary filled with cryo-protective solution (24% PEG 3350, 0.2 M KSCN, 22% glycerol and 0.1 M bis-tris propane pH 6.5). The crystal was initially loaded into a pressure cell at ambient pressure and temperature (294 K and 1 atm respectively), in which the sample was then pressurized in a pure krypton gas medium at 140 bar for 5 minutes. Then, still under pressure, the crystal was directly flash-frozen in the cell into the cold dense krypton fluid phase which acts as a coolant. Finally, the pressure was released, and the crystal was extracted from the cell and recovered in liquid nitrogen without breaking the cryogenic temperature chain. All data were collected at the ESRF ID23-1 beamline (Grenoble, France)^55^ at a wavelength of 0.861 Å (the krypton X-ray absorption edge).

### Structure determination, model building and refinement

The crystallographic data were processed using XDS^56^ for indexing and integration and Aimless^57^ for merging. Initial phases of DhaA115 were solved by molecular replacement using Phaser^58^ implemented in the Phenix package.^59^ The structure of DhaA (PDB: 4HZG) was employed as a search model for replacement in DhaA115 monomeric structures. The refinement was carried out with several automated cycles in the phenix.refine program^60^ and manual model building was performed in Coot.^61^ Crystal structures of native and krypton soaked DhaA115 were solved to resolutions of 1.6 Å and 1.55 Å in a monoclinic *P*12_1_1 space group and *P*2_1_2_1_2_1_ respectively. The final models were validated using tools provided by Coot^61^ and Molprobity.^62^ Visualization of structural data was done with PyMOL.^63^ Atomic coordinates and structure factors of the native DhaA115 and krypton-derivatized DhaA115 enzymes were deposited in the Protein Data Bank under the PDB codes 6SP5 and 6SP8.

### Small-angle X-ray scattering (SAXS)

The SAXS data sets were collected using the BioSAXS-1000, Rigaku at CEITEC (Brno, Czech Republic). Data were collected at 293 K with a focused (confocal OptiSAXS optic, Rigaku) Cu Kα X-ray (1.54 Å). The sample to detector (PILATUS 100K, Dectris) distance was 0.48 m covering a scattering vector (*q* = 4πsin(*θ*)/*λ*) range from 0.008 to 0.6 Å^−1^. Size exclusion buffer (41 mM K_2_HPO_4_, 9 mM KH_2_PO_4_, pH 7.5) was used for the blank measurement. DhaA115 protein samples were measured at concentrations of 8.5, 6.3, 4.3 and 2.2 mg ml^−1^. Six separate images were collected for buffer and sample (5 min exposure per image, 30 min total exposure). Radial averaging, data reduction and buffer subtractions were performed using SAXSLab3.0.0r1, Rigaku. Six individual scattering curves (5 min exposures) were compared to check radiation damage and averaged. Integral structural parameters were determined using PRIMUS/qt ATSAS v.2.8.4.^64^ Data points before the Guinier region were truncated. Individual scattering curves from the concentration series were manually merged for further analysis. The *ab initio* modeling for superimposition with the atomic model was performed by DAMMIN ATSAS v.2.8.4, with the computation mode set to “slow” and all other parameters kept as default. Evaluation of solution scattering and fitting to experimental scattering curves was performed using CRYSOL ATSAS v.2.8.4; automatic constant subtraction was allowed and other parameters were kept as default. Superimposition of the atomic and *ab initio* models was performed by SUPCOMB ATSAS v.2.8.4. Small-angle X-ray scattering datasets, experiment details, the atomic model and fits have been deposited in the Small Angle Scattering Biological Data Bank (www.sasbdb.org)^65^ as entry SASDHP7.

### Structural bioinformatics tools

RMSD values were calculated using pairwise structural alignment on the DALI server.^66^ Structural superposition was performed using the secondary structure matching (SSM) superimpose tool in Coot.^67^ Dimer interface and buried surface areas were calculated by the PISA tool.^17^ Analysis of residue-to-residue interactions in the crystal structure was done using Protein Interactions Calculator (PIC)^18^ with default parameters.

### Molecular dynamics simulations and analysis

The three-dimensional structures of DhaA115 were used as obtained from the X-ray diffraction analysis, for both native and krypton-soaked crystals. The solvent, crystallization molecules and krypton atoms were removed, and the double side chains were corrected to retain only the most populated conformation using the *pdb4amber* module of AmberTools 14.^68^ Hydrogen atoms were predicted using the H++ server,^69^ calculated in implicit solvent at pH 7.5, 0.1 M salinity, and internal and external dielectric constants of 10 and 80 respectively. The original crystallization solvent was added and the tLEaP program in AmberTools 14 was used to prepare the topology and coordinates files. For this, the force field ff14SB^70^ was defined, Na^+^ and Cl^−^ ions were added in order to neutralize the system and achieve a 0.10 M concentration of NaCl, and an octagonal box of TIP3P^71^ water molecules with the edges at least 10 Å away from the protein atoms was added.

The molecular dynamics (MD) simulations were carried out with the pmemd.cuda^72,73^ module of AMBER 14.^68^ In total, five minimization steps and twelve steps of equilibration dynamics were performed prior to the production MD. The first four minimization steps, composed of 2500 cycles of steepest descent followed by 7500 cycles of conjugate gradient, were performed as follows: (i) in the first step, all the atoms of the protein and ligand were restrained with 500 kcal mol^−1^ Å^−2^ harmonic force constant; (ii) in the following three, only the backbone atoms of the protein and heavy atoms of the ligand were restrained with, respectively, 500, 125, and 25 kcal mol^−1^ Å^−2^ force constant. A fifth minimization step, composed of 5000 cycles of steepest descent and 15 000 cycles of conjugate gradient, was performed without any restraints. The subsequent MD simulations employed periodic boundary conditions, the particle mesh Ewald method for treatment of the long range interactions beyond the 10 Å cutoff,^74^ the SHAKE algorithm^75^ to constrain the bonds involving the hydrogen atoms, the Langevin thermostat with collision frequency 1.0 ps^−1^, and a time step of 2 fs. Equilibration dynamics were performed in twelve steps: (i) 20 ps of gradual heating from 0 to 310 K, under constant volume, restraining the protein atoms and ligand with 200 kcal mol^−1^ Å^−2^ harmonic force constant; (ii) ten MDs of 400 ps each, at constant pressure (1 bar) and constant temperature (310 K), with gradually decreasing restraints on the backbone atoms of the protein and heavy atoms of the ligand with harmonic force constants of 150, 100, 75, 50, 25, 15, 10, 5, 1, and 0.5 kcal mol^−1^ Å^−2^; (iii) 400 ps of unrestrained MD at the same conditions as the previous restrained MDs. The energy and coordinates were saved every 10 ps. The production MDs were run for 100 ns using the same settings employed in the last equilibration step and performed in duplicate for each system.

Accelerated MD (aMD) simulations were performed for each system using the pmemd.cuda^72,73^ module of AMBER 14.^68^ The systems were prepared and minimized as previously described for the classical MDs, using the ff14SB^70^ force field. Dual energy boosts were applied to the torsional (*V*^dih^) and total potential (*V*^tot^) energy. The average dihedral (*V*_0_^dih^) and total potential (*V*_0_^tot^) energies of each system were extracted from the first 10 ns of production MD, and were used to calculate the respective energy thresholds (*E*) and acceleration factors (*α*), as previously described.^76^*E*^dih^ was set as 3.5 kcal mol^−1^ per protein residue above *V*_0_^dih^, and the corresponding acceleration factor, *α*^dih^, was set as 1/5 of that difference; the total potential energy threshold, *E*^tot^, was defined as 0.2 kcal mol^−1^ per atom of the system above *V*_0_^tot^, and the respective acceleration factor, *α*^tot^, was set as the difference between those two energies. Calculating the parameters in this way always yielded values of *E*^dih^*ca.* 27% above the respective *V*_0_^dih^. The aMDs were run without any restraints, with calculation steps of 2 fs, saving the energy and coordinate every 10 ps. These simulations were run in duplicate for 100 ns. The aMDs were performed as a complementary method to sample the conformational space equivalent to longer time scales, estimated at several orders of magnitude greater than those of the MDs (between the μs and ms time scales).^23,77^

The trajectories were analyzed using the cpptraj^78^ module of AmberTools 14, and visualized using PyMOL^63^ and VMD 1.9.1.^79^ The simulations of each type were combined to create a single one using cpptraj,^78^ and aligned to the respective crystal structures by minimizing the root-mean-square deviation (RMSD) of the backbone atoms, excluding the very flexible terminal residues of each chain (4–6 terminal residues).

### Access tunnel calculations

CAVER 3.02,^21^ was used to calculate and cluster the tunnels in the crystal structures, aggregated MD and aMD simulations of DhaA115, and the previously reported analogous simulations of DhaA^15^ and DhaA31.^76^ During the simulations the tunnels were calculated for every 10 ps-spaced snapshot using a probe radius of 0.7 Å (0.5 Å for the crystal structures of DhaA115), a shell radius of 3 Å and a shell depth of 4 Å. The starting point for the tunnel calculation was defined by the geometric center of the carboxylic oxygen atoms of the catalytic D106. The clustering was performed by the average-link hierarchical Murtagh algorithm, with a weighting coefficient of 1 and clustering threshold of 3.5 Å. Approximate clustering was allowed only when the total number of tunnels was greater than 20 000, and it was performed using 20 training clusters.

## Author contributions

K. C. and K. M. prepared the protein samples for crystallization, performed initial crystallization screenings, and optimized crystallization hits. K. M. prepared the protein samples for SAXS. K. C., P. C. and M. M. carried out krypton-derivatization of crystals and collected diffraction data. K. C., P. C. and M. M. solved the protein crystal structures. S. M. M. and D. B. performed MD analyses. M. M. and J. D. designed the project, supervised research and interpreted data. K. M. and M. M. wrote the manuscript with contributions from all authors. All authors have given approval to the final version of the manuscript.

## Data and code availability

Atomic coordinates and structural factors have been deposited in the Protein Data Bank (www.wwpdb.org) under PDB accession codes: 6SP5 and 6SP8. SAXS datasets, experiment details, atomic model and fits have been deposited in the Small Angle Scattering Biological Data Bank (www.sasbdb.org)^65^ as entry SASDHP7. Authors will release the atomic coordinates and experimental data upon article publication.

## Conflicts of interest

The authors declare no competing financial interest.

## Supplementary Material

SC-011-D0SC03367G-s001

SC-011-D0SC03367G-s002

SC-011-D0SC03367G-s003
