# Supplementary material for: Decoding the intricate network of molecular interactions of a hyperstable engineered biocatalyst
Source: Chem Sci. 2020 Sep 11;11(41):11162–78. doi: 10.1039/d0sc03367g (PMC8162949; doi:10.1039/d0sc03367g)
Supplement: SC-011-D0SC03367G-s002 [file SC-011-D0SC03367G-s002.pdf]

Electronic Supplementary Material (ESI) for Chemical Science.
This journal is © The Royal Society of Chemistry 2020

Preliminary Full wwPDB X-ray Structure Validation Report (i)

Aug 31, 2019 – 11:26 am BST

Deposition ID	:	D_1292104091
PDB ID	:	*(not yet assigned)*

This is a Preliminary Full wwPDB X-ray Structure Validation Report.

This report is produced by the wwPDB Deposition System during initial deposition but before annotation of the structure.

We welcome your comments at *validation@mail.wwpdb.org*
A user guide is available at
https://www.wwpdb.org/validation/2017/XrayValidationReportHelp
with specific help available everywhere you see the (i) symbol.

PRELIMINARY VALIDATION REPORT

The following versions of software and data (see references (i)) were used in the production of this report:

MolProbity	:	4.02b-467
Mogul	:	1.8.0 (224370), CSD as540be (2019)
Xtriage (Phenix)	:	1.13
EDS	:	2.4
buster-report	:	1.1.7 (2018)
Percentile statistics	:	20171227.v01 (using entries in the PDB archive December 27th 2017)
Refmac	:	5.8.0158
CCP4	:	7.0 (Gargrove)
Ideal geometry (proteins)	:	Engh & Huber (2001)
Ideal geometry (DNA, RNA)	:	Parkinson et al. (1996)
Validation Pipeline (wwPDB-VP)	:	2.4

1 Overall quality at a glance (i)

The following experimental techniques were used to determine the structure:
X-RAY DIFFRACTION

The reported resolution of this entry is 1.55 Å.

Percentile scores (ranging between 0-100) for global validation metrics of the entry are shown in the following graphic. The table shows the number of entries on which the scores are based.

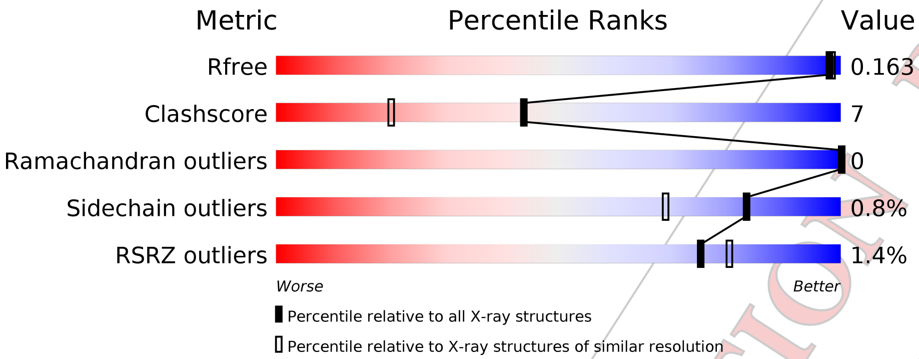

Metric	Whole archive (#Entries)	Similar resolution (#Entries, resolution range(Å))
R_{free}	111664	1224 (1.56-1.56)
Clashscore	122126	1265 (1.56-1.56)
Ramachandran outliers	120053	1240 (1.56-1.56)
Sidechain outliers	120020	1238 (1.56-1.56)
RSRZ outliers	108989	1207 (1.56-1.56)

The table below summarises the geometric issues observed across the polymeric chains and their fit to the electron density. The red, orange, yellow and green segments on the lower bar indicate the fraction of residues that contain outliers for >=3, 2, 1 and 0 types of geometric quality criteria. A grey segment represents the fraction of residues that are not modelled. The numeric value for each fraction is indicated below the corresponding segment, with a dot representing fractions <=5% The upper red bar (where present) indicates the fraction of residues that have poor fit to the electron density. The numeric value is given above the bar.

Mol	Chain	Length	Quality of chain
1	A	291	% 90% 10%
1	B	291	% 89% 11%

The following table lists non-polymeric compounds, carbohydrate monomers and non-standard residues in protein, DNA, RNA chains that are outliers for geometric or electron-density-fit criteria:

PRELIMINARY VALIDATION REPORT

Mol	Type	Chain	Res	Chirality	Geometry	Clashes	Electron density
2	KR	A	301	-	-	X	-
2	KR	A	302	-	-	X	-
2	KR	A	304	-	-	X	-
2	KR	A	307	-	-	X	-
2	KR	A	308	-	-	X	-
2	KR	A	312	-	-	X	-
2	KR	B	301	-	-	X	-
2	KR	B	302	-	-	X	-
2	KR	B	305	-	-	X	-
2	KR	B	306	-	-	X	X
2	KR	B	307	-	-	X	-
2	KR	B	308	-	-	X	-
3	SCN	B	310	-	-	X	-
5	GOL	B	317	-	-	X	-

PRELIMINARY VALIDATION REPORT

2 Entry composition (i)

There are 6 unique types of molecules in this entry. The entry contains 5574 atoms, of which 0 are hydrogens and 0 are deuteriums.

In the tables below, the ZeroOcc column contains the number of atoms modelled with zero occupancy, the AltConf column contains the number of residues with at least one atom in alternate conformation and the Trace column contains the number of residues modelled with at most 2 atoms.

- Molecule 1 is a protein.

Mol	Chain	Residues	Atoms					ZeroOcc	AltConf	Trace
1	A	291	Total 2416	C 1584	N 410	O 416	S 6	0	5	0
1	B	291	Total 2411	C 1580	N 405	O 419	S 7	0	5	0

- Molecule 2 is KRYPTON (three-letter code: KR) (formula: Kr).

Mol	Chain	Residues	Atoms		ZeroOcc	AltConf
2	A	1	Total 1	Kr 1	0	0
2	A	1	Total 1	Kr 1	0	0
2	A	1	Total 1	Kr 1	0	0
2	A	1	Total 1	Kr 1	0	0
2	A	1	Total 1	Kr 1	0	0
2	A	1	Total 1	Kr 1	0	0
2	A	1	Total 1	Kr 1	0	0
2	A	1	Total 1	Kr 1	0	0
2	A	1	Total 1	Kr 1	0	0
2	A	1	Total 1	Kr 1	0	0
2	A	1	Total 1	Kr 1	0	0
2	A	1	Total 1	Kr 1	0	0

Continued on next page...

PRELIMINARY VALIDATION REPORT

Continued from previous page...

Mol	Chain	Residues	Atoms	ZeroOcc	AltConf
2	B	1	Total Kr<br>1 1	0	0
2	B	1	Total Kr<br>1 1	0	0
2	B	1	Total Kr<br>1 1	0	0
2	B	1	Total Kr<br>1 1	0	0
2	B	1	Total Kr<br>1 1	0	0
2	B	1	Total Kr<br>1 1	0	0
2	B	1	Total Kr<br>1 1	0	0
2	B	1	Total Kr<br>1 1	0	0
2	B	1	Total Kr<br>1 1	0	0

- Molecule 3 is THIOCYANATE ION (three-letter code: SCN) (formula: CNS).

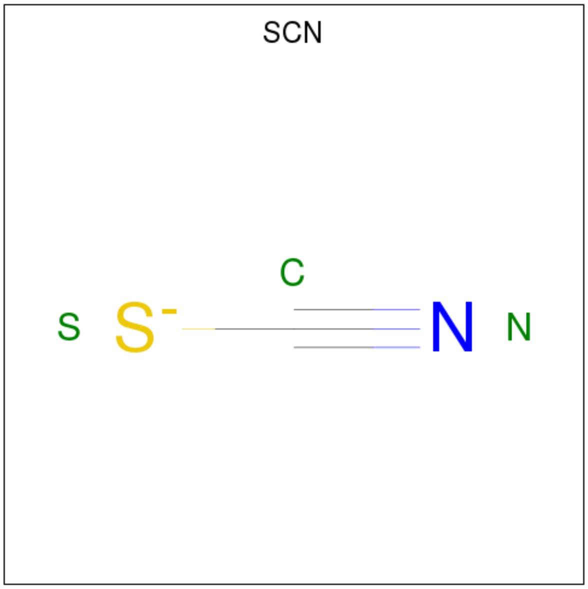

Mol	Chain	Residues	Atoms	ZeroOcc	AltConf
3	A	1	Total C N S<br>3 1 1 1	0	0
3	A	1	Total C N S<br>3 1 1 1	0	0

Continued on next page...

PRELIMINARY VALIDATION REPORT

Continued from previous page...

Mol	Chain	Residues	Atoms				ZeroOcc	AltConf
			Total	C	N	S		
3	A	1	3	1	1	1	0	0
			Total	C	N	S		
3	B	1	3	1	1	1	0	0
			Total	C	N	S		
3	B	1	3	1	1	1	0	0
			Total	C	N	S		
3	B	1	3	1	1	1	0	0

- Molecule 4 is 2-[3-(2-HYDROXY-1,1-DIHYDROXYMETHYL-ETHYLAMINO)-PROPYL AMINO]-2-HYDROXYMETHYL-PROPANE-1,3-DIOL (three-letter code: B3P) (formula: $C_{11}H_{26}N_2O_6$).

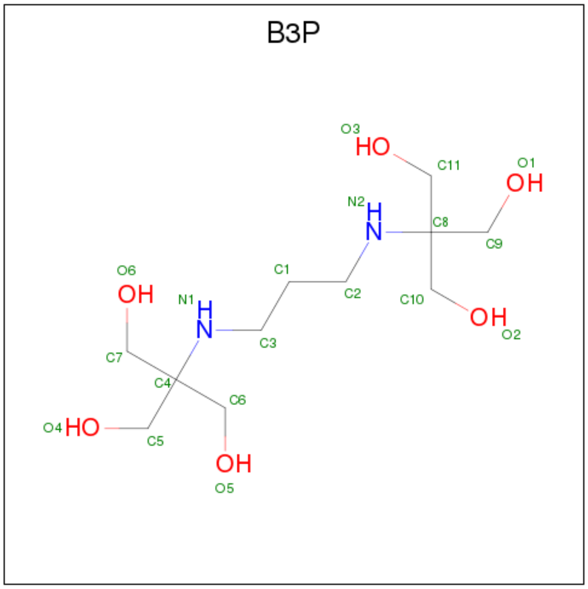

Mol	Chain	Residues	Atoms				ZeroOcc	AltConf
			Total	C	N	O		
4	A	1	19	11	2	6	0	0
			Total	C	N	O		
4	B	1	19	11	2	6	0	0

- Molecule 5 is GLYCEROL (three-letter code: GOL) (formula: $C_3H_8O_3$).

PRELIMINARY VALIDATION REPORT

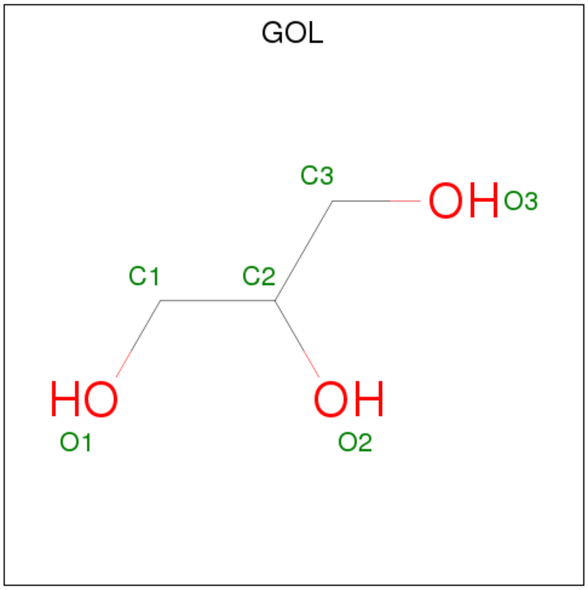

Mol	Chain	Residues	Atoms	ZeroOcc	AltConf
5	A	1	Total C O<br>6 3 3	0	0
5	A	1	Total C O<br>6 3 3	0	0
5	B	1	Total C O<br>6 3 3	0	0
5	B	1	Total C O<br>6 3 3	0	0
5	B	1	Total C O<br>6 3 3	0	0
5	B	1	Total C O<br>6 3 3	0	0
5	B	1	Total C O<br>6 3 3	0	0
5	B	1	Total C O<br>6 3 3	0	0

- Molecule 6 is water.

Mol	Chain	Residues	Atoms	ZeroOcc	AltConf
6	A	319	Total O<br>323 323	0	4
6	B	298	Total O<br>299 299	0	1

PRELIMINARY VALIDATION REPORT

3 Residue-property plots ⓘ

These plots are drawn for all protein, RNA and DNA chains in the entry. The first graphic for a chain summarises the proportions of the various outlier classes displayed in the second graphic. The second graphic shows the sequence view annotated by issues in geometryand electron density. Residues are color-coded according to the number of geometric quality criteria for which they contain at least one outlier: green = 0, yellow = 1, orange = 2 and red = 3 or more. A red dot above a residue indicates a poor fit to the electron density (RSRZ > 2). Stretches of 2 or more consecutive residues without any outlier are shown as a green connector. Residues present in the sample, but not in the model, are shown in grey.

- Molecule 1:

Chain A:
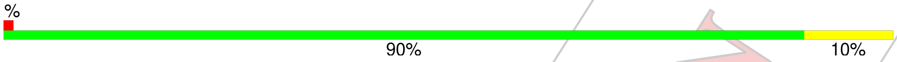

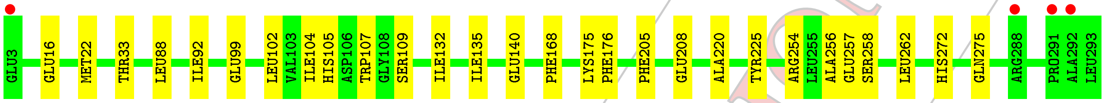

- Molecule 1:

Chain B:
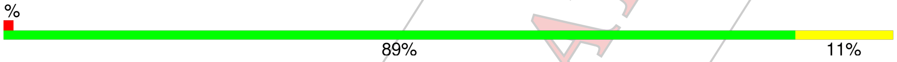

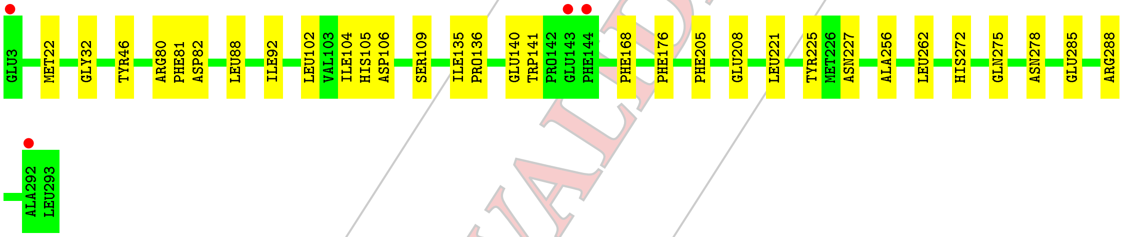

PRELIMINARY VALIDATION REPORT

4 Data and refinement statistics (i)

Property	Value	Source
Space group	P 21 21 21	Depositor
Cell constants<br>a, b, c, α, β, γ	67.98Å 82.05Å 144.18Å<br>90.00° 90.00° 90.00°	Depositor
Resolution (Å)	49.20 – 1.55<br>49.20 – 1.55	Depositor<br>EDS
% Data completeness<br>(in resolution range)	98.8 (49.20-1.55)<br>98.8 (49.20-1.55)	Depositor<br>EDS
R_{merge}	0.07	Depositor
R_{sym}	(Not available)	Depositor
$< I/\sigma(I) >$ [1]	3.13 (at 1.55Å)	Xtriage
Refinement program	PHENIX	Depositor
R, R_{free}	0.160 , 0.181<br>0.161 , 0.163	Depositor<br>DCC
R_{free} test set	5763 reflections (4.97%)	wwPDB-VP
Wilson B-factor (Å^2)	18.3	Xtriage
Anisotropy	0.839	Xtriage
Bulk solvent k_{sol}(e/Å^3), B_{sol}(Å^2)	0.39 , 48.5	EDS
L-test for twinning[2]	$< \|L\| >= 0.53$, $< L^2 >= 0.37$	Xtriage
Estimated twinning fraction	No twinning to report.	Xtriage
F_o,F_c correlation	0.98	EDS
Total number of atoms	5574	wwPDB-VP
Average B, all atoms (Å^2)	21.0	wwPDB-VP

Xtriage's analysis on translational NCS is as follows: *The analyses of the Patterson function reveals a significant off-origin peak that is 24.94 % of the origin peak, indicating pseudo-translational symmetry. The chance of finding a peak of this or larger height randomly in a structure without pseudo-translational symmetry is equal to 3.4808e-03. The detected translational NCS is most likely also responsible for the elevated intensity ratio.*

[1] Intensities estimated from amplitudes.

[2] Theoretical values of $< |L| >$, $< L^2 >$ for acentric reflections are 0.5, 0.333 respectively for untwinned datasets, and 0.375, 0.2 for perfectly twinned datasets.

PRELIMINARY VALIDATION REPORT

5 Model quality (i)

5.1 Standard geometry (i)

Bond lengths and bond angles in the following residue types are not validated in this section: GOL, SCN, B3P, KR

The Z score for a bond length (or angle) is the number of standard deviations the observed value is removed from the expected value. A bond length (or angle) with $|Z| > 5$ is considered an outlier worth inspection. RMSZ is the root-mean-square of all Z scores of the bond lengths (or angles).

Mol	Chain	Bond lengths		Bond angles	
		RMSZ	$\#\|Z\| > 5$	RMSZ	$\#\|Z\| > 5$
1	A	0.34	0/2504	0.53	0/3426
1	B	0.35	0/2499	0.54	0/3421
All	All	0.34	0/5003	0.54	0/6847

There are no bond length outliers.

There are no bond angle outliers.

There are no chirality outliers.

There are no planarity outliers.

5.2 Too-close contacts (i)

In the following table, the Non-H and H(model) columns list the number of non-hydrogen atoms and hydrogen atoms in the chain respectively. The H(added) column lists the number of hydrogen atoms added and optimized by MolProbity. The Clashes column lists the number of clashes within the asymmetric unit, whereas Symm-Clashes lists symmetry related clashes.

Mol	Chain	Non-H	H(model)	H(added)	Clashes	Symm-Clashes
1	A	2416	0	2359	27	0
1	B	2411	0	2344	36	0
2	A	12	0	0	19	0
2	B	9	0	0	25	0
3	A	9	0	0	1	0
3	B	9	0	0	2	0
4	A	19	0	26	0	0
4	B	19	0	26	0	0
5	A	12	0	16	0	0
5	B	36	0	48	8	0
6	A	323	0	0	4	0

Continued on next page...

PRELIMINARY VALIDATION REPORT

Continued from previous page...

Mol	Chain	Non-H	H(model)	H(added)	Clashes	Symm-Clashes
6	B	299	0	0	3	0
All	All	5574	0	4819	70	0

The all-atom clashscore is defined as the number of clashes found per 1000 atoms (including hydrogen atoms). The all-atom clashscore for this structure is 7.

All (70) close contacts within the same asymmetric unit are listed below, sorted by their clash magnitude.

Atom-1	Atom-2	Interatomic distance (Å)	Clash overlap (Å)
2:A:303:KR:KR	2:A:304:KR:KR	2.94	1.08
2:B:302:KR:KR	2:B:303:KR:KR	3.03	0.98
1:B:221:LEU:HB3	2:B:307:KR:KR	2.40	0.83
1:A:208:GLU:HG3	2:A:308:KR:KR	2.42	0.80
1:B:208[A]:GLU:OE2	2:B:306:KR:KR	2.63	0.77
1:A:135:ILE:HD12	2:A:307:KR:KR	2.49	0.74
1:A:16:GLU:HG2	6:A:570:HOH:O	1.88	0.73
1:B:256:ALA:HA	1:B:262:LEU:HD23	1.69	0.72
1:B:81:PHE:HD1	2:B:306:KR:KR	2.38	0.67
1:A:254[A]:ARG:NH2	6:A:401:HOH:O	2.21	0.66
1:B:205:PHE:CE1	2:B:306:KR:KR	3.10	0.66
2:B:306:KR:KR	6:B:411:HOH:O	2.76	0.65
1:A:175:LYS:NZ	6:A:404:HOH:O	2.30	0.64
1:B:135:ILE:HD12	2:B:308:KR:KR	2.59	0.64
1:B:82:ASP:HB3	5:B:317:GOL:H32	1.82	0.61
1:B:88:LEU:HD13	2:B:305:KR:KR	2.61	0.61
1:B:22:MET:CE	2:B:305:KR:KR	3.11	0.59
1:A:88:LEU:HD13	2:A:301:KR:KR	2.64	0.58
1:B:81:PHE:HA	2:B:306:KR:KR	2.65	0.57
1:B:221:LEU:CB	2:B:307:KR:KR	3.13	0.57
1:B:205:PHE:CD1	2:B:306:KR:KR	3.20	0.56
1:B:82:ASP:CB	5:B:317:GOL:H32	2.36	0.55
1:A:22:MET:CE	2:A:301:KR:KR	3.15	0.55
1:B:140:GLU:O	2:B:308:KR:KR	2.85	0.55
1:A:205:PHE:CD1	2:A:308:KR:KR	3.21	0.55
1:B:227[A]:ASN:ND2	6:B:407:HOH:O	2.38	0.55
1:A:22:MET:HE1	2:A:301:KR:KR	2.68	0.54
1:B:22:MET:HE1	2:B:305:KR:KR	2.69	0.54
1:B:81:PHE:HB2	2:B:306:KR:KR	2.69	0.54
1:A:88:LEU:CD1	2:A:301:KR:KR	3.18	0.52
1:B:46:TYR:HB2	5:B:319:GOL:H32	1.90	0.52
1:B:81:PHE:CD1	2:B:306:KR:KR	3.22	0.52

Continued on next page...

PRELIMINARY VALIDATION REPORT

Continued from previous page...

Atom-1	Atom-2	Interatomic distance (Å)	Clash overlap (Å)
1:A:33:THR:HG23	1:A:99[B]:GLU:HG2	1.91	0.52
1:B:136:PRO:HG2	1:B:140:GLU:OE1	2.09	0.52
1:B:22:MET:HE2	2:B:305:KR:KR	2.71	0.52
1:B:205:PHE:HE1	2:B:306:KR:KR	2.54	0.52
1:B:88:LEU:CD1	2:B:305:KR:KR	3.19	0.52
1:A:140:GLU:O	2:A:307:KR:KR	2.89	0.51
5:B:319:GOL:H31	6:B:605:HOH:O	2.09	0.51
1:B:104:ILE:HD12	1:B:109:SER:HA	1.93	0.51
1:A:254[B]:ARG:HG3	2:A:312:KR:KR	2.75	0.47
1:A:256:ALA:HA	1:A:262:LEU:HD23	1.96	0.47
2:B:302:KR:KR	3:B:310:SCN:N	3.08	0.47
1:A:102:LEU:HD21	2:A:302:KR:KR	2.76	0.47
2:A:304:KR:KR	3:A:313:SCN:N	3.09	0.46
1:A:254[B]:ARG:CG	2:A:312:KR:KR	3.25	0.45
1:B:81:PHE:CA	2:B:306:KR:KR	3.25	0.45
1:B:81:PHE:CB	2:B:306:KR:KR	3.26	0.44
1:A:258:SER:HB3	2:A:312:KR:KR	2.78	0.44
1:B:92:ILE:HD11	2:B:301:KR:KR	2.79	0.44
1:B:285:GLU:OE2	1:B:288:ARG:NH1	2.50	0.44
1:B:141:TRP:HA	2:B:308:KR:KR	2.79	0.43
1:A:175:LYS:HA	1:A:175:LYS:HD3	1.75	0.43
1:A:22:MET:HE2	2:A:301:KR:KR	2.79	0.43
1:B:176:PHE:HB3	1:B:272:HIS:HB3	2.01	0.43
1:A:92:ILE:HD11	2:A:302:KR:KR	2.80	0.42
1:B:105:HIS:CG	1:B:275:GLN:HE22	2.37	0.42
1:A:176:PHE:HB3	1:A:272:HIS:HB3	2.01	0.42
1:B:80:ARG:HE	5:B:317:GOL:C3	2.33	0.41
1:A:257:GLU:HB3	2:A:312:KR:KR	2.81	0.41
1:B:106:ASP:OD1	3:B:310:SCN:N	2.54	0.41
1:A:104:ILE:HD12	1:A:109:SER:HA	2.02	0.41
1:B:278:ASN:OD1	5:B:318:GOL:H11	2.21	0.41
1:A:107:TRP:HA	1:A:132[B]:ILE:HD12	2.03	0.41
1:A:105:HIS:CG	1:A:275:GLN:HE22	2.39	0.41
2:A:312:KR:KR	6:A:469:HOH:O	2.99	0.41
1:B:32:GLY:O	5:B:316:GOL:H11	2.21	0.41
1:A:220:ALA:HB2	5:B:317:GOL:H2	2.04	0.40
1:A:254[B]:ARG:HG2	2:A:312:KR:KR	2.82	0.40
1:B:102:LEU:HD21	2:B:301:KR:KR	2.83	0.40

There are no symmetry-related clashes.

PRELIMINARY VALIDATION REPORT

5.3 Torsion angles ⓘ

5.3.1 Protein backbone ⓘ

In the following table, the Percentiles column shows the percent Ramachandran outliers of the chain as a percentile score with respect to all X-ray entries followed by that with respect to entries of similar resolution.

The Analysed column shows the number of residues for which the backbone conformation was analysed, and the total number of residues.

Mol	Chain	Analysed	Favoured	Allowed	Outliers	Percentiles	
1	A	294/291 (101%)	284 (97%)	10 (3%)	0	100	100
1	B	294/291 (101%)	283 (96%)	11 (4%)	0	100	100
All	All	588/582 (101%)	567 (96%)	21 (4%)	0	100	100

There are no Ramachandran outliers to report.

5.3.2 Protein sidechains ⓘ

In the following table, the Percentiles column shows the percent sidechain outliers of the chain as a percentile score with respect to all X-ray entries followed by that with respect to entries of similar resolution.

The Analysed column shows the number of residues for which the sidechain conformation was analysed, and the total number of residues.

Mol	Chain	Analysed	Rotameric	Outliers	Percentiles	
1	A	256/251 (102%)	254 (99%)	2 (1%)	83	69
1	B	256/251 (102%)	254 (99%)	2 (1%)	83	69
All	All	512/502 (102%)	508 (99%)	4 (1%)	83	69

All (4) residues with a non-rotameric sidechain are listed below:

Mol	Chain	Res	Type
1	A	168	PHE
1	A	225	TYR
1	B	168	PHE
1	B	225	TYR

Some sidechains can be flipped to improve hydrogen bonding and reduce clashes. There are no such sidechains identified.

PRELIMINARY VALIDATION REPORT

5.3.3 RNA (i)

There are no RNA molecules in this entry.

5.4 Non-standard residues in protein, DNA, RNA chains (i)

There are no non-standard protein/DNA/RNA residues in this entry.

5.5 Carbohydrates (i)

There are no carbohydrates in this entry.

5.6 Ligand geometry (i)

Of 37 ligands modelled in this entry, 21 are monoatomic - leaving 16 for Mogul analysis.

In the following table, the Counts columns list the number of bonds (or angles) for which Mogul statistics could be retrieved, the number of bonds (or angles) that are observed in the model and the number of bonds (or angles) that are defined in the Chemical Component Dictionary. The Link column lists molecule types, if any, to which the group is linked. The Z score for a bond length (or angle) is the number of standard deviations the observed value is removed from the expected value. A bond length (or angle) with $|Z| > 2$ is considered an outlier worth inspection. RMSZ is the root-mean-square of all Z scores of the bond lengths (or angles).

Mol	Type	Chain	Res	Link	Bond lengths			Bond angles		
					Counts	RMSZ	$\#\|Z\| > 2$	Counts	RMSZ	$\#\|Z\| > 2$
3	SCN	A	313	-	1,2,2	1.12	0	0,1,1	0.00	-
3	SCN	A	314	-	1,2,2	0.56	0	0,1,1	0.00	-
3	SCN	A	315	-	1,2,2	0.35	0	0,1,1	0.00	-
4	B3P	A	316	-	18,18,18	0.60	0	21,23,23	1.31	1 (4%)
5	GOL	A	317	-	5,5,5	0.53	0	5,5,5	0.32	0
5	GOL	A	318	-	5,5,5	0.52	0	5,5,5	0.27	0
3	SCN	B	310	-	1,2,2	1.48	0	0,1,1	0.00	-
3	SCN	B	311	-	1,2,2	0.47	0	0,1,1	0.00	-
3	SCN	B	312	-	1,2,2	0.36	0	0,1,1	0.00	-
4	B3P	B	313	-	18,18,18	0.63	0	21,23,23	1.07	2 (9%)
5	GOL	B	314	-	5,5,5	0.51	0	5,5,5	0.19	0
5	GOL	B	315	-	5,5,5	0.54	0	5,5,5	0.37	0
5	GOL	B	316	-	5,5,5	0.46	0	5,5,5	0.73	0
5	GOL	B	317	-	5,5,5	0.48	0	5,5,5	0.33	0
5	GOL	B	318	-	5,5,5	0.52	0	5,5,5	0.30	0
5	GOL	B	319	-	5,5,5	0.61	0	5,5,5	0.37	0

PRELIMINARY VALIDATION REPORT

In the following table, the Chirals column lists the number of chiral outliers, the number of chiral centers analysed, the number of these observed in the model and the number defined in the Chemical Component Dictionary. Similar counts are reported in the Torsion and Rings columns. '-' means no outliers of that kind were identified.

Mol	Type	Chain	Res	Link	Chirals	Torsions	Rings
4	B3P	A	316	-	-	2/28/28/28	-
5	GOL	A	317	-	-	4/4/4/4	-
5	GOL	A	318	-	-	0/4/4/4	-
4	B3P	B	313	-	-	0/28/28/28	-
5	GOL	B	314	-	-	0/4/4/4	-
5	GOL	B	315	-	-	0/4/4/4	-
5	GOL	B	316	-	-	0/4/4/4	-
5	GOL	B	317	-	-	2/4/4/4	-
5	GOL	B	318	-	-	2/4/4/4	-
5	GOL	B	319	-	-	3/4/4/4	-

There are no bond length outliers.

All (3) bond angle outliers are listed below:

Mol	Chain	Res	Type	Atoms	Z	Observed(^{o})	Ideal(^{o})
4	A	316	B3P	C2-N2-C8	-3.88	110.61	116.12
4	B	313	B3P	C2-N2-C8	-2.28	112.88	116.12
4	B	313	B3P	C3-N1-C4	-2.21	112.99	116.12

There are no chirality outliers.

All (13) torsion outliers are listed below:

Mol	Chain	Res	Type	Atoms
5	B	319	GOL	C1-C2-C3-O3
5	B	317	GOL	C1-C2-C3-O3
5	A	317	GOL	O1-C1-C2-C3
5	A	317	GOL	C1-C2-C3-O3
5	B	318	GOL	O1-C1-C2-C3
5	B	319	GOL	O2-C2-C3-O3
5	B	317	GOL	O2-C2-C3-O3
5	A	317	GOL	O2-C2-C3-O3
5	B	318	GOL	O1-C1-C2-O2
5	B	319	GOL	O1-C1-C2-C3
4	A	316	B3P	C10-C8-C9-O1
5	A	317	GOL	O1-C1-C2-O2
4	A	316	B3P	N2-C8-C9-O1

PRELIMINARY VALIDATION REPORT

There are no ring outliers.

4 monomers are involved in 8 short contacts:

Mol	Chain	Res	Type	Clashes	Symm-Clashes
5	B	316	GOL	1	0
5	B	317	GOL	4	0
5	B	318	GOL	1	0
5	B	319	GOL	2	0

The following is a two-dimensional graphical depiction of Mogul quality analysis of bond lengths, bond angles, torsion angles, and ring geometry for all instances of the Ligand of Interest. In addition, ligands with molecular weight > 250 and outliers as shown on the validation Tables will also be included. For torsion angles, if less then 5% of the Mogul distribution of torsion angles is within 10 degrees of the torsion angle in question, then that torsion angle is considered an outlier. Any bond that is central to one or more torsion angles identified as an outlier by Mogul will be highlighted in the graph. For rings, the root-mean-square deviation (RMSD) between the ring in question and similar rings identified by Mogul is calculated over all ring torsion angles. If the average RMSD is greater than 60 degrees and the minimal RMSD between the ring in question and any Mogul-identified rings is also greater than 60 degrees, then that ring is considered an outlier. The outliers are highlighted in purple. The color gray indicates Mogul did not find sufficient equivalents in the CSD to analyse the geometry.

Ligand B3P A 316

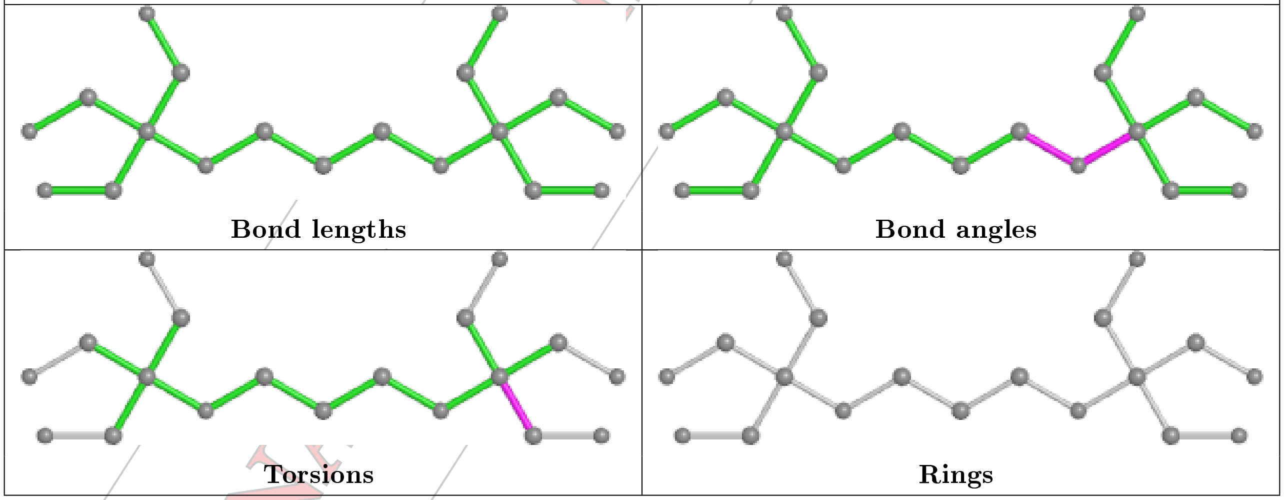

PRELIMINARY VALIDATION REPORT

Ligand B3P B 313

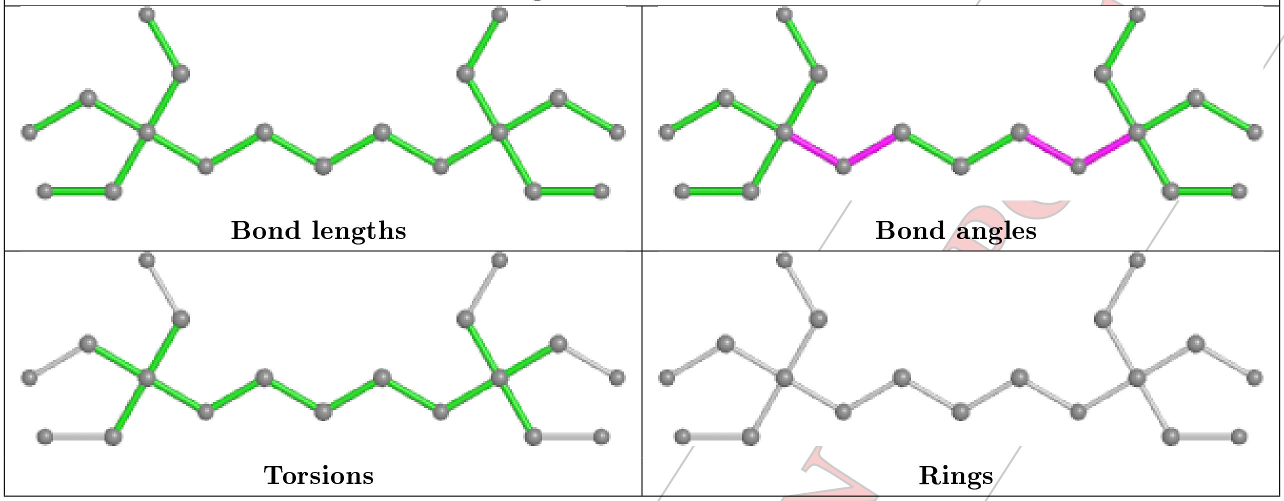

5.7 Other polymers (i)

There are no such residues in this entry.

5.8 Polymer linkage issues (i)

There are no chain breaks in this entry.

PRELIMINARY VALIDATION

6 Fit of model and data (i)

6.1 Protein, DNA and RNA chains (i)

In the following table, the column labelled '#RSRZ> 2' contains the number (and percentage) of RSRZ outliers, followed by percent RSRZ outliers for the chain as percentile scores relative to all X-ray entries and entries of similar resolution. The OWAB column contains the minimum, median, 95th percentile and maximum values of the occupancy-weighted average B-factor per residue. The column labelled 'Q< 0.9' lists the number of (and percentage) of residues with an average occupancy less than 0.9.

Mol	Chain	Analysed	<RSRZ>	#RSRZ>2			OWAB($Å^2$)	Q<0.9
1	A	291/291 (100%)	-0.21	4 (1%)	75	80	12, 17, 30, 51	0
1	B	291/291 (100%)	-0.23	4 (1%)	75	80	12, 18, 31, 52	0
All	All	582/582 (100%)	-0.22	8 (1%)	75	80	12, 18, 31, 52	0

All (8) RSRZ outliers are listed below:

Mol	Chain	Res	Type	RSRZ
1	B	3	GLU	4.6
1	B	292	ALA	3.4
1	A	3	GLU	3.0
1	B	144	PHE	2.9
1	B	143	GLU	2.6
1	A	292	ALA	2.4
1	A	288	ARG	2.2
1	A	291	PRO	2.1

6.2 Non-standard residues in protein, DNA, RNA chains (i)

There are no non-standard protein/DNA/RNA residues in this entry.

6.3 Carbohydrates (i)

There are no carbohydrates in this entry.

6.4 Ligands (i)

In the following table, the Atoms column lists the number of modelled atoms in the group and the number defined in the chemical component dictionary. The B-factors column lists the minimum,

PRELIMINARY VALIDATION REPORT

median, 95th percentile and maximum values of B factors of atoms in the group. The column labelled 'Q< 0.9' lists the number of atoms with occupancy less than 0.9.

Mol	Type	Chain	Res	Atoms	RSCC	RSR	B-factors($Å^2$)	Q<0.9
2	KR	B	306	1/?	0.49	0.49	19,19,19,19	1
2	KR	B	307	1/?	0.71	0.38	25,25,25,25	1
5	GOL	A	317	6/?	0.72	0.20	36,43,46,48	0
4	B3P	A	316	19/?	0.78	0.15	19,24,37,45	19
5	GOL	B	317	6/?	0.78	0.22	33,35,40,45	0
2	KR	A	308	1/?	0.81	0.36	25,25,25,25	1
4	B3P	B	313	19/?	0.81	0.14	19,25,41,45	19
5	GOL	B	318	6/?	0.83	0.26	23,35,41,49	0
5	GOL	A	318	6/?	0.83	0.26	24,36,47,54	0
5	GOL	B	319	6/?	0.84	0.32	30,34,38,41	0
2	KR	A	312	1/?	0.85	0.15	36,36,36,36	1
2	KR	B	308	1/?	0.85	0.17	27,27,27,27	1
2	KR	A	307	1/?	0.86	0.19	30,30,30,30	1
5	GOL	B	314	6/?	0.87	0.12	25,26,35,35	0
5	GOL	B	316	6/?	0.87	0.33	22,37,42,42	0
5	GOL	B	315	6/?	0.90	0.23	31,34,38,44	0
2	KR	B	302	1/?	0.91	0.17	24,24,24,24	1
2	KR	A	304	1/?	0.91	0.21	23,23,23,23	1
2	KR	B	309	1/?	0.95	0.13	30,30,30,30	1
3	SCN	B	312	3/?	0.95	0.25	39,39,47,57	0
2	KR	B	304	1/?	0.96	0.10	22,22,22,22	1
2	KR	A	311	1/?	0.97	0.07	28,28,28,28	1
2	KR	A	305	1/?	0.98	0.07	35,35,35,35	1
2	KR	A	302	1/?	0.98	0.14	16,16,16,16	1
2	KR	B	305	1/?	0.98	0.10	18,18,18,18	1
2	KR	B	303	1/?	0.98	0.07	23,23,23,23	1
3	SCN	B	311	3/?	0.98	0.10	22,22,33,37	3
2	KR	A	310	1/?	0.98	0.08	21,21,21,21	1
2	KR	B	301	1/?	0.98	0.08	15,15,15,15	1
2	KR	A	303	1/?	0.98	0.08	22,22,22,22	1
3	SCN	A	315	3/?	0.98	0.21	28,28,31,40	0
3	SCN	A	313	3/?	0.99	0.07	18,18,20,25	0
3	SCN	A	314	3/?	0.99	0.12	21,21,34,37	3
3	SCN	B	310	3/?	0.99	0.09	18,18,20,24	0
2	KR	A	306	1/?	0.99	0.13	19,19,19,19	1
2	KR	A	301	1/?	0.99	0.14	18,18,18,18	1
2	KR	A	309	1/?	0.99	0.07	19,19,19,19	1

The following is a graphical depiction of the model fit to experimental electron density of all instances of the Ligand of Interest. In addition, ligands with molecular weight > 250 and outliers as shown on the geometry validation Tables will also be included. Each fit is shown from different

PRELIMINARY VALIDATION REPORT

orientation to approximate a three-dimensional view.

Electron density around B3P A 316:

$2mF_o$-DF_c (at 0.7 rmsd) in gray
mF_o-DF_c (at 3 rmsd) in purple (negative)
and green (positive)

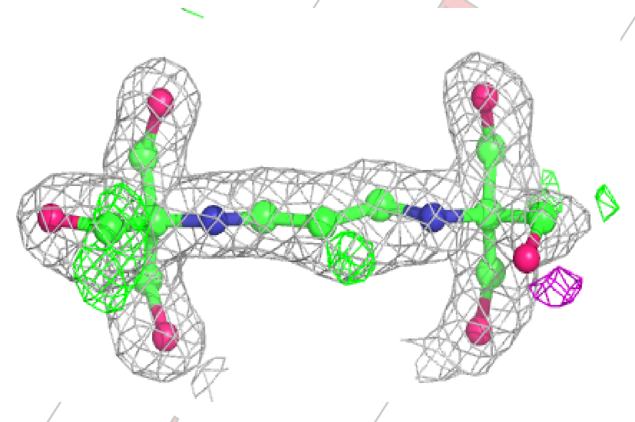

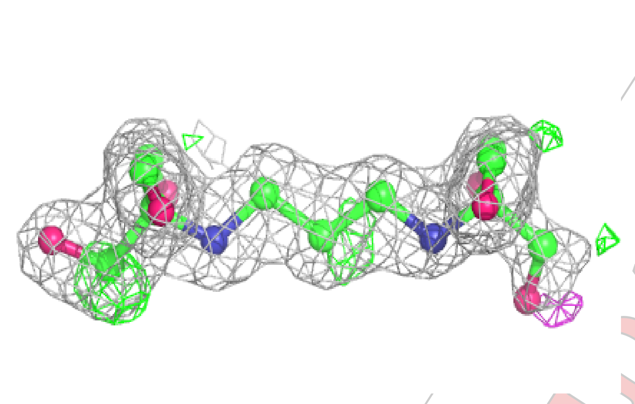

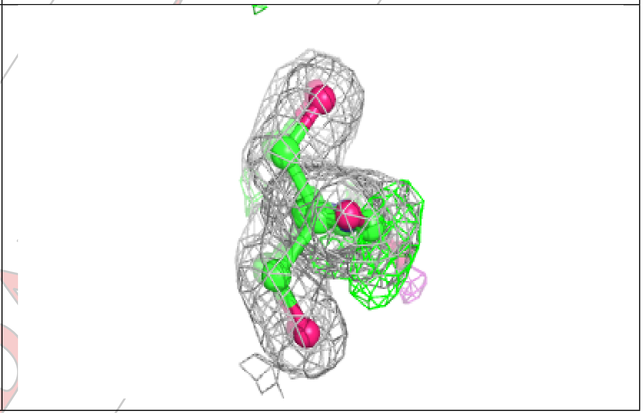

Electron density around B3P B 313:

$2mF_o$-DF_c (at 0.7 rmsd) in gray
mF_o-DF_c (at 3 rmsd) in purple (negative)
and green (positive)

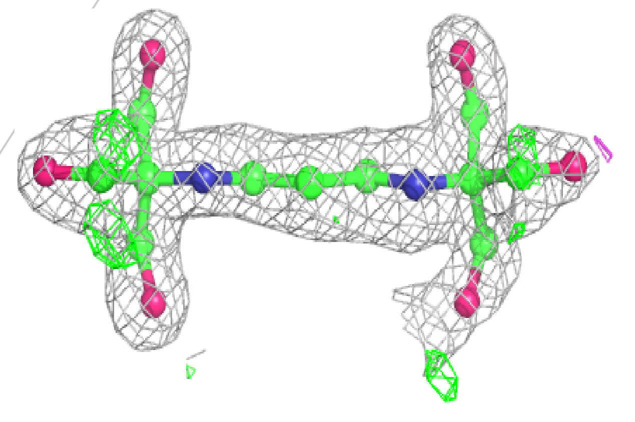

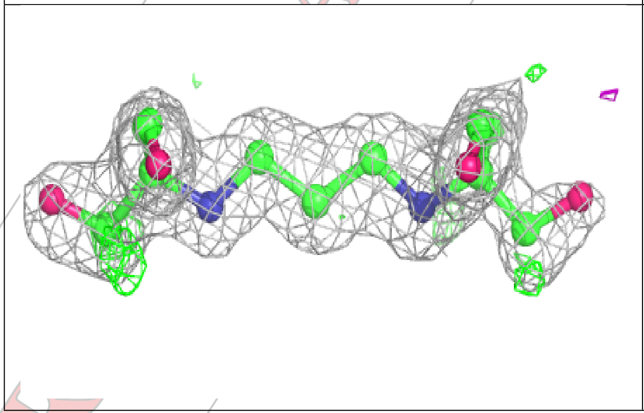

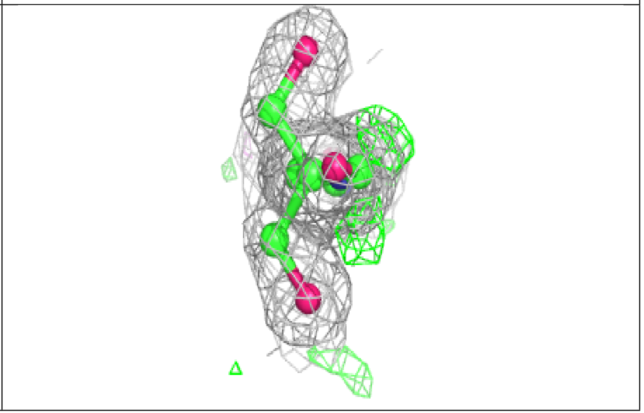

6.5 Other polymers (i)

There are no such residues in this entry.

PRELIMINARY VALIDATION REPORT
