# Supplementary material for: Decoding the intricate network of molecular interactions of a hyperstable engineered biocatalyst
Source: Chem Sci. 2020 Sep 11;11(41):11162–78. doi: 10.1039/d0sc03367g (PMC8162949; doi:10.1039/d0sc03367g)
Supplement: SC-011-D0SC03367G-s003 [file SC-011-D0SC03367G-s003.pdf]

Electronic Supplementary Material (ESI) for Chemical Science.
This journal is © The Royal Society of Chemistry 2020

SUPPLEMENTARY DATA

Decoding the Intricate Network of Molecular Interactions of a Hyperstable Engineered Biocatalyst

Klara Markova[1,2,‡], Klaudia Chmelova[1,2,‡], Sergio M. Marques[1,2], Philippe Carpentier[3,4], David Bednar[1,2], Jiri Damborsky[1,2,*], Martin Marek[1,2*]

1. Loschmidt Laboratories, Department of Experimental Biology and RECETOX, Faculty of Science, Masaryk University, Kamenice 5, 625 00 Brno, Czech Republic
2. International Clinical Research Center, St. Anne's University Hospital Brno, Pekarska 53, 656 91 Brno, Czech Republic
3. Université Grenoble Alpes, CNRS, CEA, Interdisciplinary Research Institute of Grenoble (IRIG), Laboratoire Chimie et Biologie des Métaux (LCBM), 17 avenue des Martyrs, 38054 Grenoble, France
4. European Synchrotron Radiation Facility (ESRF), 71 Avenue des Martyrs, 38043 Grenoble, France

‡ Joint first authors

* Corresponding authors: jiri@chemi.muni.cz; martin.marek@recetox.muni.cz

Table of contents

Figure S1. Cartoon representation of the two molecules (A and B) present in the asymmetric unit of the crystal of DhaA115.

Figure S2. Structural comparison of DhaA115 and DhaA. Superposition of the engineered hyperstable DhaA115 (blue) and native DhaA (green) structures.

Figure S3. Structural comparison of DhaA and DhaA115 crystal structures showing the tilt of α9 helix.

Figure S4. Structural features of DhaA115 krypton crystal derivative.

Figure S5. Superposition of the DhaA115 krypton derivative (blue) and native DhaA115 (violet) structures.

Figure S6. Root-mean square deviation (RMSD) of DhaA and DhaA115 during the dynamics simulations, for: A) MD simulations, and B) aMD simulations.

Figure S7. B-factors of the backbone atoms of DhaA115 and DhaA.

Table S1. Average RMSD and distances measured in the crystal structures, MD and aMD simulations of DhaA115 and DhaA.

Table S2. Properties of the top-ranked tunnels calculated in the crystal structures, MD and aMD simulations of DhaA115 and two other DhaA variants.

Figure S8. Access tunnels calculated in the crystal structure of DhaA115 (A), krypton-soaked DhaA115 (B), wild-type DhaA (PDB ID 4E46) (C), and DhaA31 (PDB-ID 3RK4) (D).

Figure S9. Access tunnels calculated in the MD and aMD simulations of DhaA115 (A and B) and DhaA (C and D).

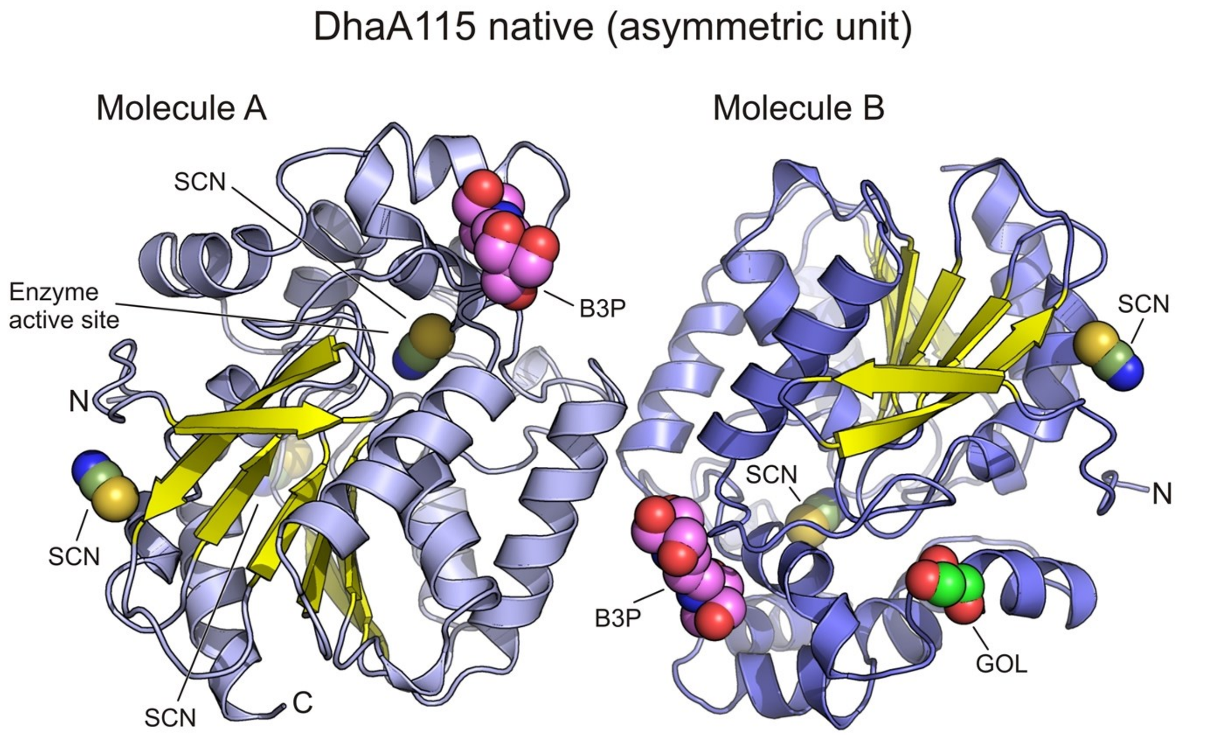

Figure S1. Cartoon representation of the two molecules (A and B) present in the asymmetric unit of the crystal of DhaA115. Bis-tris propane (B3P), isothiocyanate (SCN) and glycerol (GOL) molecules bound to the enzyme are ligands represented as violet, dark green and green spheres, respectively.

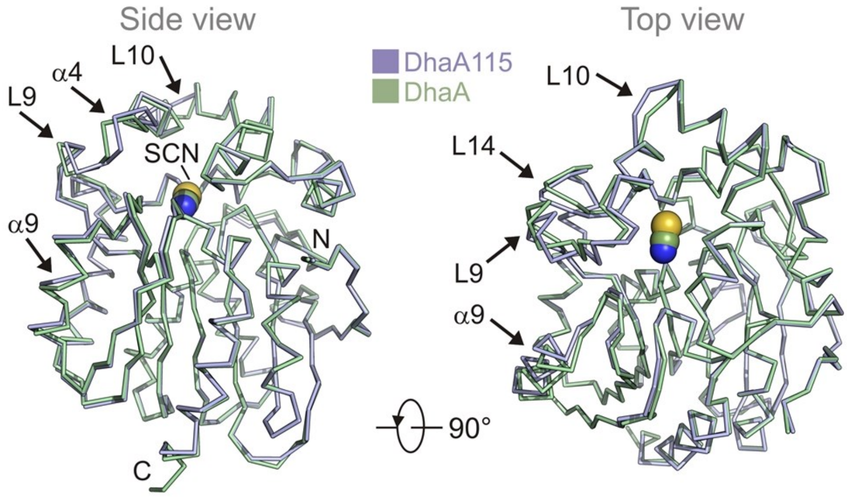

Figure S2. Structural comparison of DhaA115 and DhaA. Superposition of the engineered hyperstable DhaA115 (blue) and native DhaA (green) structures. Both enzymes adopt the same fold, but several specific backbone changes are observed in L9, L10 and L14 loops and α4 and α9 helices. Isothiocyanate (SCN) molecule bound in the DhaA115 enzyme active site is shown as spheres.

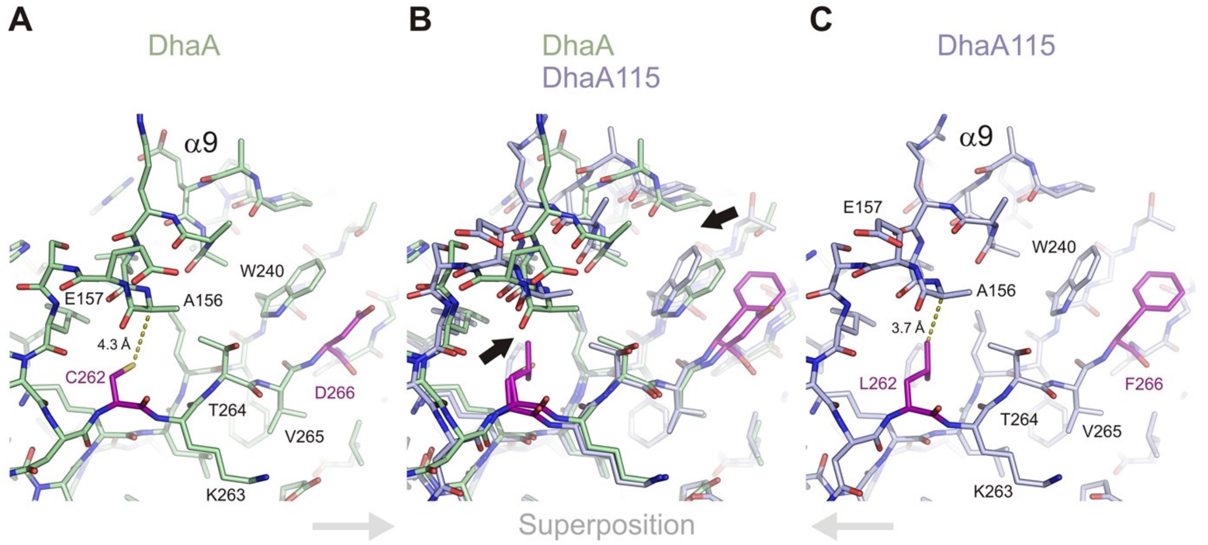

Figure S3. Structural comparison of DhaA and DhaA115 crystal structures showing the tilt of α9 helix. Stick representations of DhaA (left panel), DhaA115 (right panel) and their superposition (middle panel). The stabilizing mutations (C262L and D266F) are shown as purple sticks. Distances between A156 and C262 (A), and A156 and L262 (C) are depicted by yellow dashed lines. Black arrows depict the slight lift of the α9 helix induced by presence the both L262 and F266 residues.

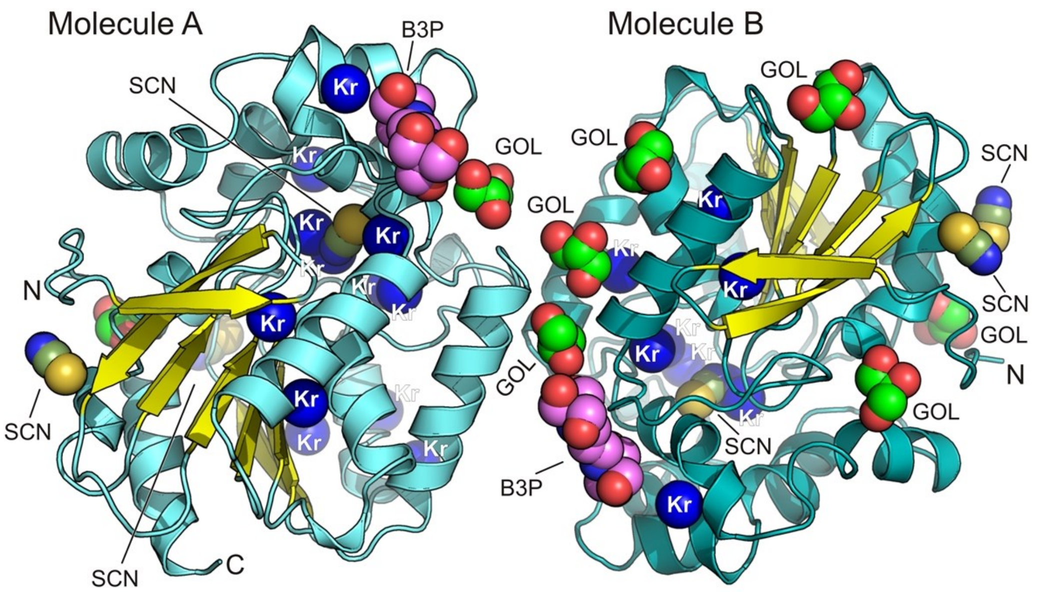

Figure S4. Structural features of DhaA115 krypton crystal derivative. The two non-crystallographic DhaA115 molecules (A and B) that are observed in the asymmetric unit are represented as cartoon. Krypton atoms (Kr) are shown as blue spheres; Bis-tris propane (B3P), isothiocyanate (SCN) and glycerol (GOL) molecules bound to the enzyme are shown as violet (B3P), dark green (SCN) and green (GOL) spheres respectively.

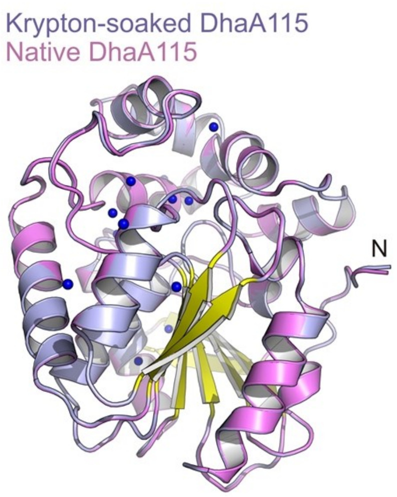

Figure S5. Superposition of the DhaA115 krypton derivative (blue) and native DhaA115 (violet) structures. Note that the krypton pressurization induced no protein backbone change. The krypton atoms bound to DhaA115 enzyme are shown as blue spheres.

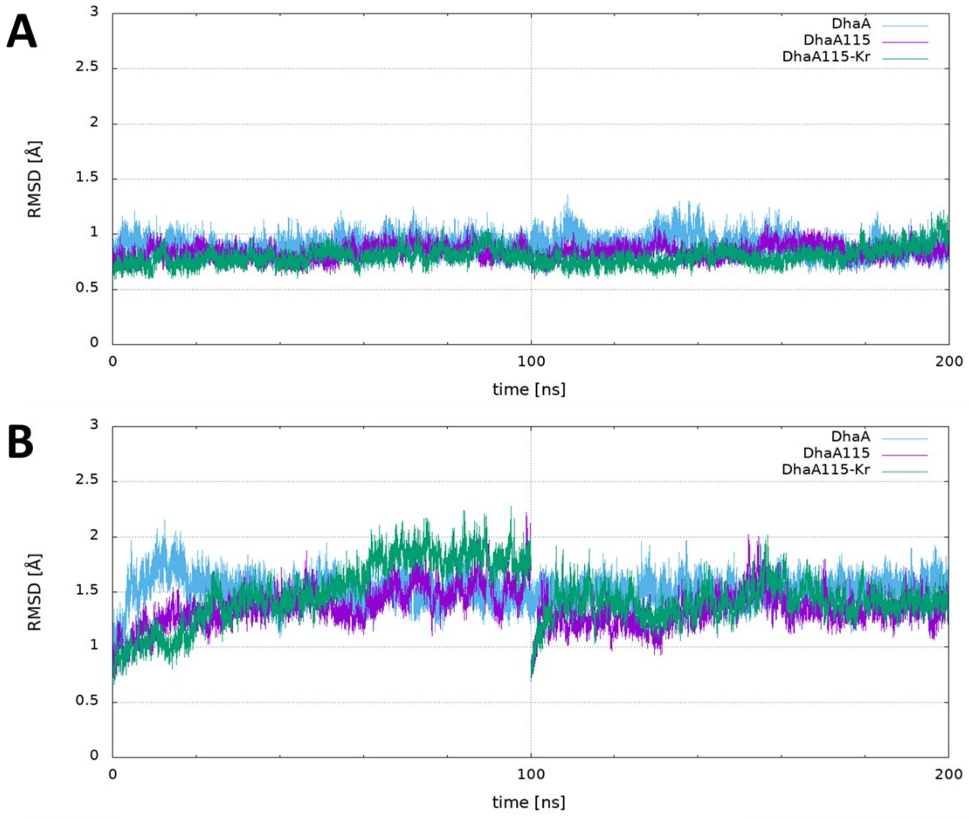

Figure S6. Root-mean square deviation (RMSD) of DhaA and DhaA115 during the dynamics simulations, for: A) MD simulations, and B) aMD simulations. The "DhaA115" lines correspond to the simulations started from the original crystal structure, and "DhaA115–Kr" started from the structure obtained from the krypton-soaked crystals. The RMSD was calculated for the backbone atoms excluding the 4 terminal residues with highest flexibility; in the case of DhaA115, the plots represent an aggregate of two independent simulations of 100 ns each, as used in all further analyses.

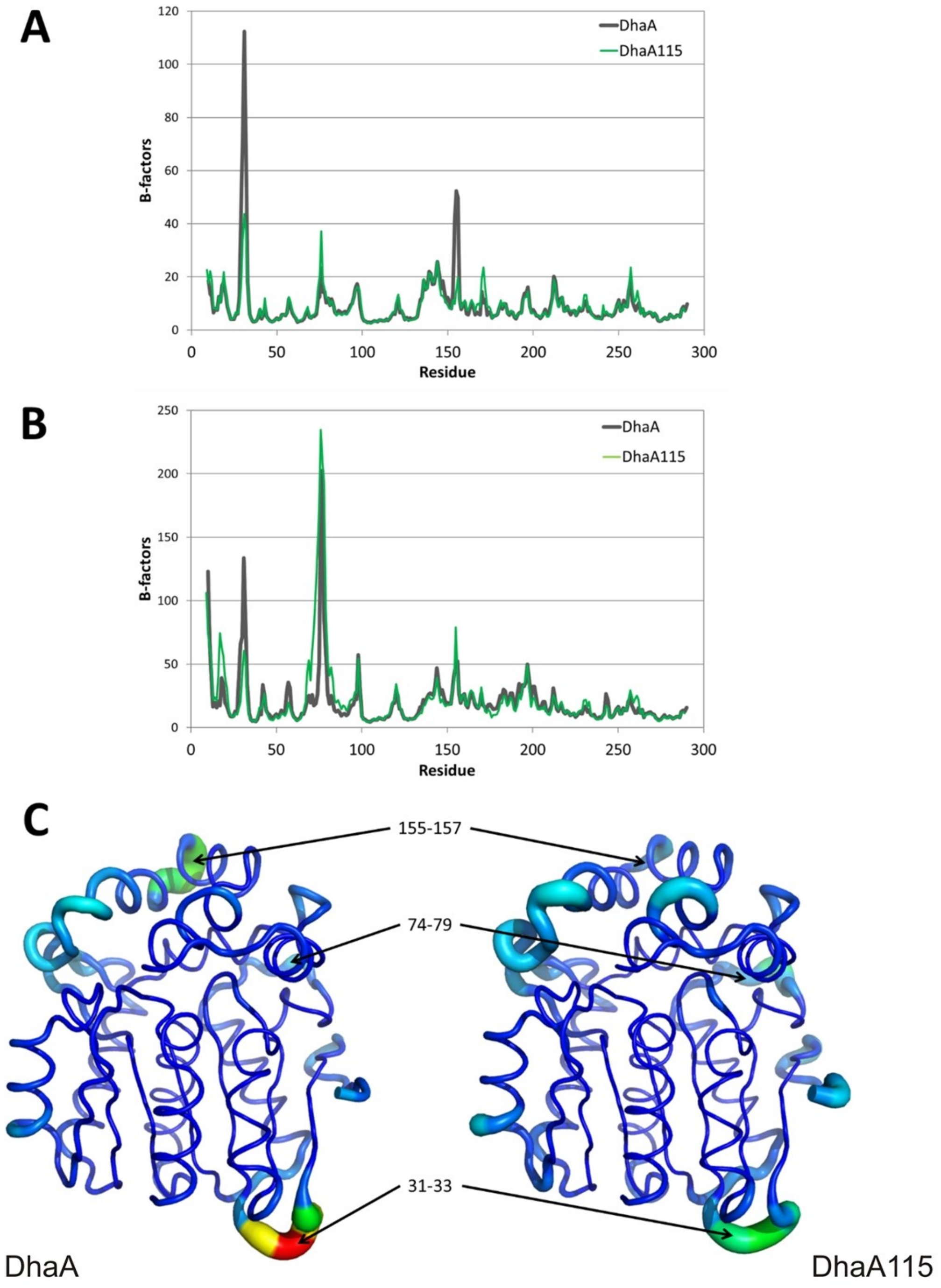

Figure S7. B-factors of the backbone atoms of DhaA115 and DhaA. (A) MD, (B) aMD simulations, and (C) putty tube representation of the B-factors of the backbone atoms, calculated from the MD simulations of DhaA (left) and DhaA115 (right).

Table S1. Average RMSD and distances measured in the crystal structures, MD and aMD simulations of DhaA115 and DhaA.[a]

RMSD/distances (Å)	Label	DhaA115 crystal	DhaA115 MD	DhaA115 aMD	DhaA crystal	DhaA MD	DhaA aMD
RMSD protein (5-289@ backbone)		0	0.77 ± 0.07	1.31 ± 0.18	0	0.85 ± 0.09	1.43 ± 0.15
RMSD W/V219@ backbone		0	0.62 ± 0.24	0.84 ± 0.28	0	0.62 ± 0.23	0.75 ± 0.27
RMSD W/V219@ heavy atoms		0	0.69 ± 0.24	0.88 ± 0.29	0	0.70 ± 0.25	0.82 ± 0.29
dist (W/V219@ca – P136@ca)	**d1**	10.04	10.41 ± 0.28	10.40 ± 0.30	8.75	9.85 ± 0.50	9.96 ± 0.52
dist (W/V219@ca – P134@ca)	**d2**	6.08	6.31 ± 0.30	6.31 ± 0.32	6.08	6.16 ± 0.37	6.12 ± 0.46
dist (W219@ch2 – P136@ca)	**d3**	4.03	4.30 ± 0.27	4.34 ± 0.30	-	-	-

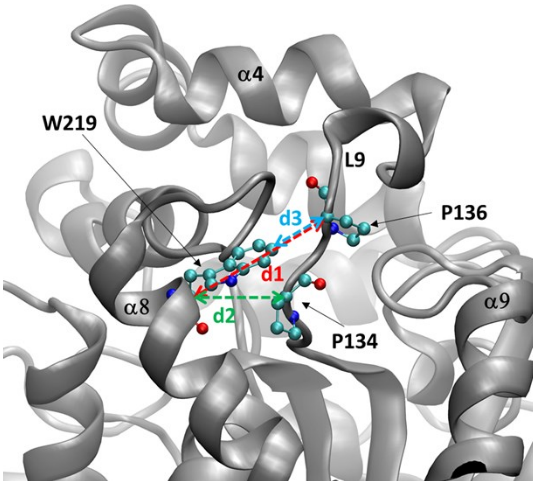

RMSD/distances (Å)	Label	DhaA115 crystal	DhaA115 MD	DhaA115 aMD	DhaA crystal	DhaA MD	DhaA aMD
dist (F/D266@ca - W240@ca)	**d4**	5.12	5.15 ± 0.15	5.15 ± 0.17	5.34	5.37 ± 0.19	5.32 ± 0.24
dist (P249@ca - W240@ca)	**d5**	7.32	7.35 ± 0.37	7.43 ± 0.44	6.81	6.84 ± 0.38	7.04 ± 0.49
dist (F/D266@ca - P249@ca)	**d6**	9.28	9.40 ± 0.36	9.35 ± 0.48	8.66	8.71 ± 0.39	8.92 ± 0.47

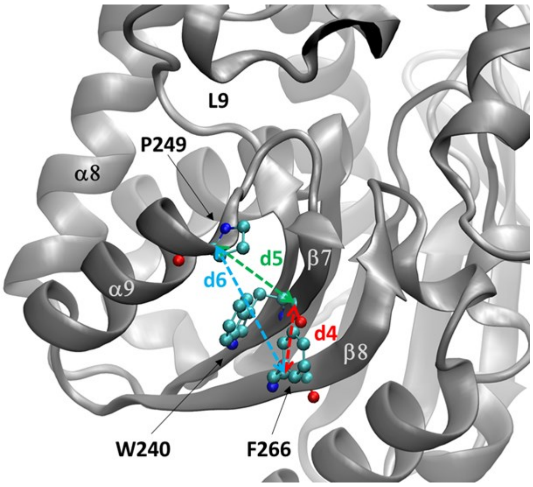

dist (R133@cz - E140@cd)	**d7**	4.58	4.66 ± 0.72	4.50 ± 0.50	8.58	4.70 ± 0.93	4.49 ± 0.59
dist (R133@cz - E251@cd)	**d8**	4.16	4.22 ± 0.40	4.11 ± 0.37	4.58	4.24 ± 0.42	4.15 ± 0.41
dist (E251@cd - R254@cz)	**d9**	4.41	4.10 ± 0.24	4.34 ± 0.88	4.96	4.11 ± 0.41	4.49 ± 1.26
dist (E140@ca - L246@ca)	**d10**	8.40	8.29 ± 0.48	8.40 ± 0.45	9.18	8.83 ± 0.60	8.52 ± 0.54

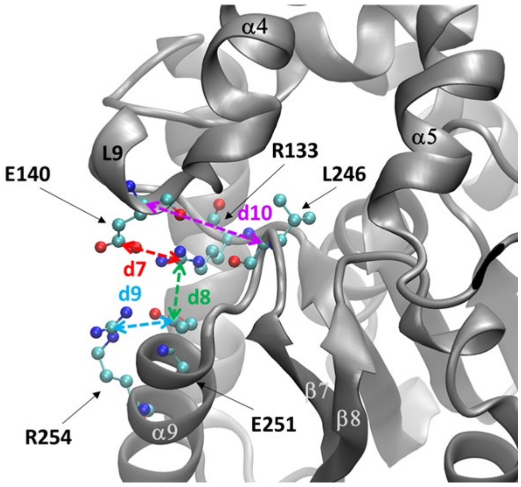

[a]Values calculated over 20,000 snapshots from the simulations (spaced by 10 ps), corresponding to a total simulation time of 200 ns; the RMSD values were computed having the respective crystal structures as reference; the average values are reported with the respective standard deviations; the figures depict the residues of DhaA115 and the distances referred in each section of the table.

Table S2. Properties of the top-ranked tunnels calculated in the crystal structures, MD and aMD simulations of DhaA115 and two other DhaA variants.[a]

	Tunnel No.	Standard name[b]	% Frames	Avg. BR ± SD (Å)	Max. BR (Å)	Avg. L ± SD (Å)	Priority
Crystals							
DhaA115[c]	1	p2b	n.a.	0.59	n.a.	18.45	0.223
	2	p1a	n.a.	0.68	n.a.	18.51	0.192
	3	p3	n.a.	0.51	n.a.	17.36	0.097
DhaA115-Kr[c, d]	1	p2b	n.a.	0.71	n.a.	17.28	0.356
	2	p1a	n.a.	0.58	n.a.	17.97	0.240
	3	p4	n.a.	0.67	n.a.	23.88	0.098
DhaAwt[c]	1	p1	n.a.	1.43	n.a.	14.97	0.636
	2	p2a	n.a.	0.91	n.a.	19.28	0.451
DhaA31[c]	1	p1	n.a.	0.97	n.a.	14.50	0.455
	2	p1a	n.a.	0.79	n.a.	17.72	0.372
	3	p2a	n.a.	0.71	n.a.	18.47	0.209
MDs							
DhaA115	1	p2b	60.0	0.84 ± 0.11	1.34	18.6 ± 2.2	0.194
	2	p3a	50.3	0.77 ± 0.07	1.17	22.8 ± 3.7	0.102
	3	p1	32.2	0.80 ± 0.10	1.41	17.7 ± 2.3	0.092
	4	p1a	27.0	0.77 ± 0.07	1.16	19.0 ± 2.1	0.071
DhaA115-Kr[d]	1	p2b	78.4	0.84 ± 0.11	1.37	18.5 ± 2.7	0.260
	2	p1	33.6	0.79 ± 0.09	1.56	17.8 ± 2.7	0.096
	3	p1a	31.2	0.78 ± 0.08	1.41	18.4 ± 2.2	0.087
	4	p3a	41.5	0.78 ± 0.07	1.20	22.6 ± 3.7	0.087
DhaAwt[e]	1	p1	99.4	1.15 ± 0.20	1.73	15.7 ± 1.8	0.517
	2	p2b	72.2	0.87 ± 0.12	1.55	18.4 ± 2.2	0.267
	3	p3	32.3	0.80 ± 0.08	1.27	16.7 ± 2.2	0.098
DhaA31[e]	1	p1	70.3	0.84 ± 0.13	1.68	14.7 ± 2.0	0.262
	2	p3	28.9	0.77 ± 0.07	1.14	17.1 ± 2.5	0.078
	3	p2b	20.6	0.78 ± 0.08	1.3	17.3 ± 2.5	0.062
aMDs							
DhaA115	1	p4	56.3	0.94 ± 0.21	1.69	21.4 ± 5.5	0.193
	2	p1	34.6	0.82 ± 0.13	1.61	18.4 ± 3.1	0.102
	3	p3	33.2	0.77 ± 0.07	1.33	19.5 ± 3.7	0.083
	4	p1a	30.6	0.78 ± 0.08	1.51	19.7 ± 3.3	0.078
	5	p2b	29.0	0.78 ± 0.09	1.38	20.1 ± 3.6	0.077
DhaA115-Kr	1	p4	42.6	1.01 ± 0.24	1.70	21.2 ± 6.0	0.156
	2	p1	34.0	0.81 ± 0.12	1.72	18.8 ± 3.1	0.095
	3	p1a	30.3	0.78 ± 0.09	1.48	19.7 ± 3.0	0.077
	4	p2b	28.2	0.78 ± 0.08	1.33	20.1 ± 3.6	0.075
DhaAwt[e]	1	p1	96.6	1.20 ± 0.25	1.73	15.7 ± 2.1	0.518
	2	p1a	55.8	0.94 ± 0.21	1.72	17.3 ± 2.4	0.225
	3	p3	32.5	0.78 ± 0.08	1.71	17.5 ± 2.7	0.094
	4	p2b	28.5	0.79 ± 0.09	1.71	19.8 ± 3.0	0.084
DhaA31[e]	1	p1	70.6	0.86 ± 0.15	1.72	15.8 ± 2.3	0.267
	2	p2b	47.9	0.81 ± 0.09	1.39	18.1 ± 3.2	0.152
	3	p1a	24.6	0.78 ± 0.08	1.51	16.9 ± 2.2	0.079
	4	p3	27.5	0.77 ± 0.07	1.25	18.2 ± 2.9	0.072

[a]"Tunnel No." is the tunnel ranking by importance, "% Frames" is the percentage of snapshots with detected tunnel (with minimum radius of 0.7 Å in general, or 0.5 Å for the crystal structures of DhaA115), "Avg. BR" is

the average bottleneck radius over the simulations (Å), "Max. BR" is the highest bottleneck radius observed during the entire simulation (Å), "Avg. L" is the average tunnel length (Å). The tunnels are raked according to their *priority*, which quantifies their potential importance in the transport of ligands, and combines their average radius, length, curvature, and the frequency with which they were detected during the simulation,[1]; n.a. stands for "not applicable"; the average values are reported with the respective standard deviations.

[b]Tunnel names according to ref.[2]

[c]Tunnels calculated in the crystal structures protonated as prepared for the MD simulations.

[d]The *DhaA115-Kr* entries correspond to the structure or simulations obtained from soaking the crystals of DhaA115 in krypton, which were analyzed and simulated after removing the Kr atoms

[e]Tunnels from single simulations performed under similar conditions as DhaA115 and published before[3]

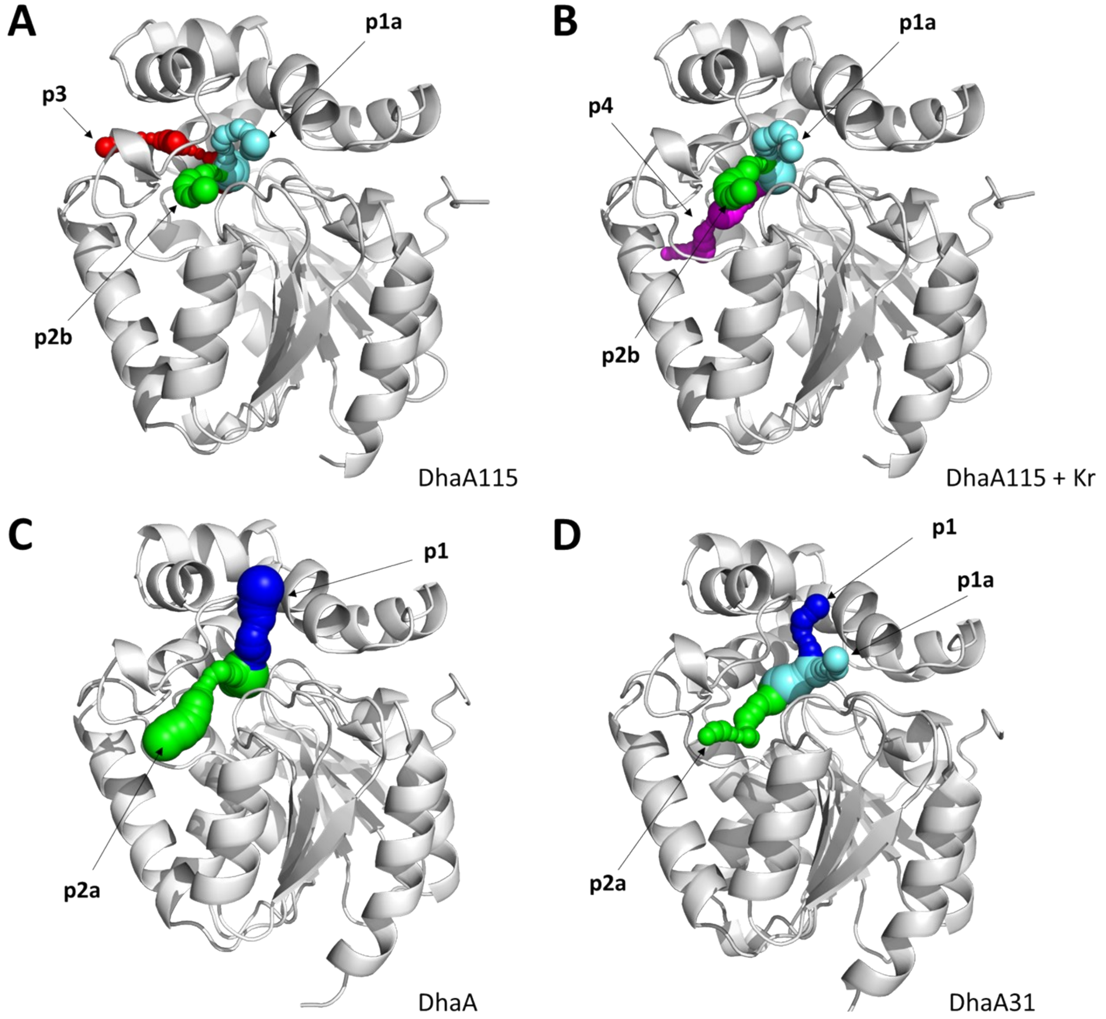

Figure S8. Access tunnels calculated in the crystal structure of DhaA115 (A), krypton-soaked DhaA115 (B), wild-type DhaA (PDB ID 4E46) (C), and DhaA31 (PDB-ID 3RK4) (D). The tunnels are represented by the colored spheres and are labeled according to the standard nomenclature of the tunnels found in the DhaA variants[2].

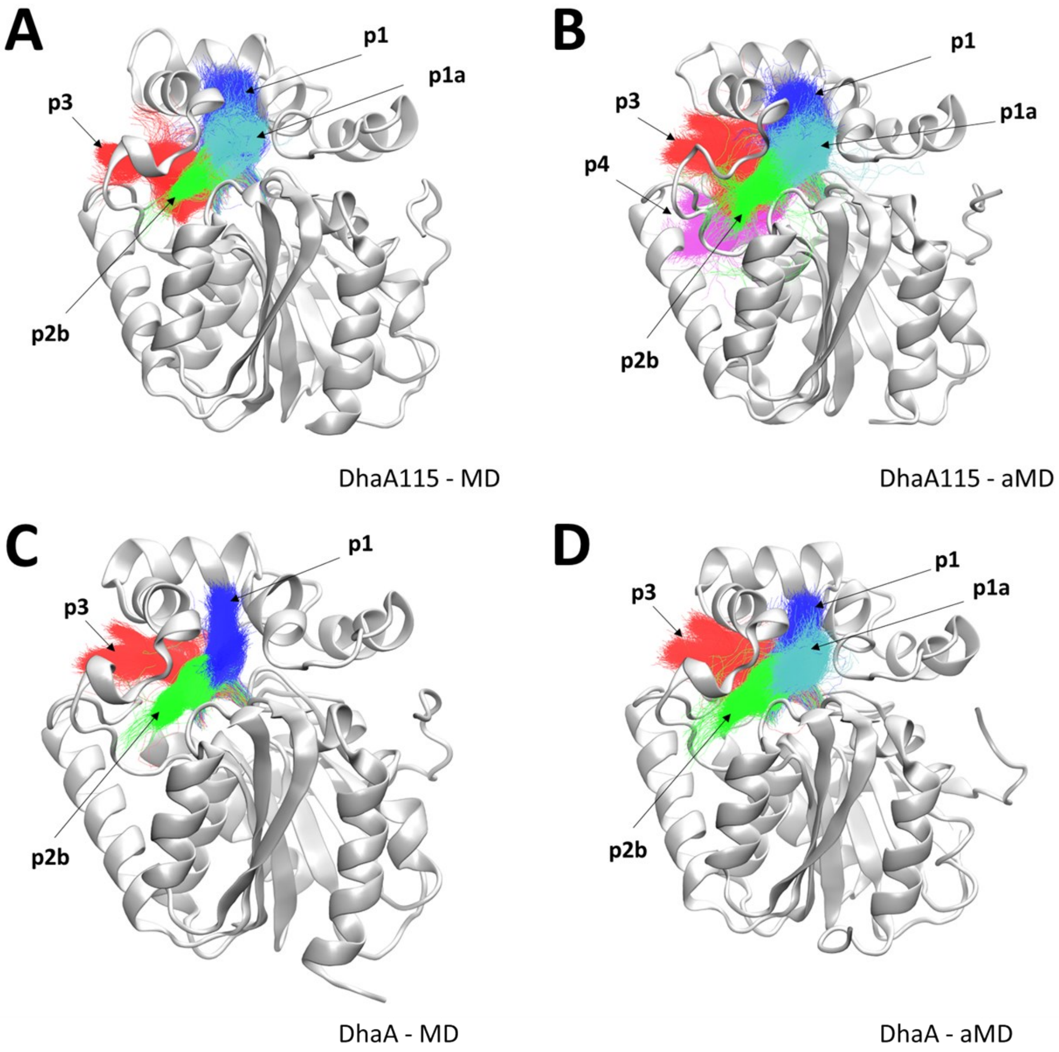

Figure S9. Access tunnels calculated in the MD and aMD simulations of DhaA115 (A and B) and DhaA (C and D). The tunnels are represented by the superimposed colored lines, collected from all the simulation snapshots and clustered according to their topologies, and are labeled according to the standard nomenclature of the tunnels found in the DhaA variants[2].

References

(1) Chovancova, E.; Pavelka, A.; Benes, P.; Strnad, O.; Brezovsky, J.; Kozlikova, B.; Gora, A.; Sustr, V.; Klvana, M.; Medek, P.; Biedermannova, L.; Sochor, J.; Damborsky, J. CAVER 3.0: A Tool for the Analysis of Transport Pathways in Dynamic Protein Structures. *PLoS Comput. Biol.* **2012**, *8* (10), e1002708.

(2) Klvana, M.; Pavlova, M.; Koudelakova, T.; Chaloupkova, R.; Dvorak, P.; Prokop, Z.; Stsiapanava, A.; Kuty, M.; Kuta-Smatanova, I.; Dohnalek, J.; Kulhanek, P.; Wade, R. C.; Damborsky, J. Pathways and Mechanisms for Product Release in the Engineered Haloalkane Dehalogenases Explored Using Classical and Random Acceleration Molecular Dynamics Simulations. *J. Mol. Biol.* **2009**, *392* (5), 1339–1356.

(3) Marques, S. M.; Dunajova, Z.; Prokop, Z.; Chaloupkova, R.; Brezovsky, J.; Damborsky, J. Catalytic Cycle of Haloalkane Dehalogenases toward Unnatural Substrates Explored by Computational Modeling. *J. Chem. Inf. Model.* **2017**, *57* (8), 1970–1989.
